# Para$^2$: parameterized path reduction, acceleration, and SMT for reachability in threshold-guarded distributed algorithms

**DOI:** 10.1007/s10703-017-0297-4

**Published:** 2017-09-20

**Authors:** Igor Konnov, Marijana Lazić, Helmut Veith, Josef Widder

**Affiliations:** Institute of Information Systems E184/4, TU Wien (Vienna University of Technology), Favoritenstraße 9-11, 1040 Vienna, Austria

**Keywords:** Parameterized verification, Bounded model checking, Completeness, Partial orders in distributed systems, Reduction, Fault-tolerant distributed algorithms, Byzantine faults

## Abstract

Automatic verification of threshold-based fault-tolerant distributed algorithms (FTDA) is challenging: FTDAs have multiple parameters that are restricted by arithmetic conditions, the number of processes and faults is parameterized, and the algorithm code is parameterized due to conditions counting the number of received messages. Recently, we introduced a technique that first applies data and counter abstraction and then runs bounded model checking (BMC). Given an FTDA, our technique computes an upper bound on the diameter of the system. This makes BMC complete for reachability properties: it always finds a counterexample, if there is an actual error. To verify state-of-the-art FTDAs, further improvement is needed. In contrast to encoding bounded executions of a counter system over an abstract finite domain in SAT, in this paper, we encode bounded executions over integer counters in SMT. In addition, we introduce a new form of reduction that exploits acceleration and the structure of the FTDAs. This aggressively prunes the execution space to be explored by the solver. In this way, we verified safety of seven FTDAs that were out of reach before.

## Introduction

Replication is a classic approach to make computing systems more reliable. In order to avoid a single point of failure, one uses multiple processes in a distributed system. Then, if some of these processes fail (e.g., by crashing or deviating from their expected behavior) the distributed system as a whole should stay operational. For this purpose one uses fault-tolerant distributed algorithms (FTDAs). These algorithms have been extensively studied in distributed computing theory [1, 50], and found application in safety critical systems (automotive or aeronautic industry). With the recent advent of data centers and cloud computing we observe growing interest in fault-tolerant distributed algorithms, and their correctness, also for more mainstream computer science applications [19, 20, 31, 47, 52, 54, 60].

We consider automatic verification techniques specifically for threshold-based fault-tolerant distributed algorithms. In these algorithms, processes collect messages from their peers, and check whether the number of received messages reaches a threshold, e.g., a threshold may ensure that acknowledgments from a majority of processes have been received. Waiting for majorities, or more generally waiting for quorums, is a key pattern of many fault-tolerant algorithms, e.g., consensus, replicated state machine, and atomic commit. In [34] we introduced an efficient encoding of these algorithms, which we used in [33] for abstraction-based parameterized model checking of safety and liveness of several case study algorithms, which are parameterized in the number of processes *n* and the fraction of faults *t*, e.g., $n>3t$. In [41] we were able to verify reachability properties of more involved algorithms by applying bounded model checking. We showed how to make bounded model checking complete in the parameterized case. In particular, we considered counter systems where we record for each local state, how many processes are in this state. We have one counter per local state $\ell$, denoted by $\kappa [\ell ]$. A process step from local state $\ell$ to local state $\ell '$ is modeled by decrementing $\kappa [\ell ]$ and incrementing $\kappa [\ell ']$. When $\delta$ processes perform the same step one after the other, we allow the processes to do the *accelerated step* that instantaneously changes the two affected counters by $\delta$. The number $\delta$ is called *acceleration factor*, which can vary in a single run.

As we focus on threshold-based FTDAs, we consider counter systems defined by *threshold automata*. Here, transitions are guarded by *threshold guards* that compare a shared integer variable to a linear combination of parameters, e.g., $x\ge n-t$ or $x<t$, where *x* is a shared variable and *n* and *t* are parameters.

Completeness of the method [41] with respect to reachability is shown by proving a bound on the diameter of the accelerated system. Inspired by Lamport’s view of distributed computation as partial order on events [43], our method uses a reduction similar to Lipton’s [48]. Instead of pruning executions that are “similar” to ones explored before as in *partial order reduction* [28, 53, 59], we use the partial order to show (offline) that every run has a similar run of bounded length. Interestingly, the bound is independent of the parameters. In [41], we introduced the following automated method, which combines this idea with data abstraction [33]:

1. Apply a parametric data abstraction to the process code to get a finite state process description, and construct the threshold automaton (TA) [33, 36].
2. Compute the diameter bound, based on the control flow of the TA.
3. Construct a system with abstract counters, i.e., a counter abstraction [33, 55].
4. Perform SAT-based bounded model checking [6, 16] up to the diameter bound, to check whether bad states are reached in the counter abstraction.
5. If a counterexample is found, check its feasibility and refine, if needed [13, 33].

**Fig. 1:**
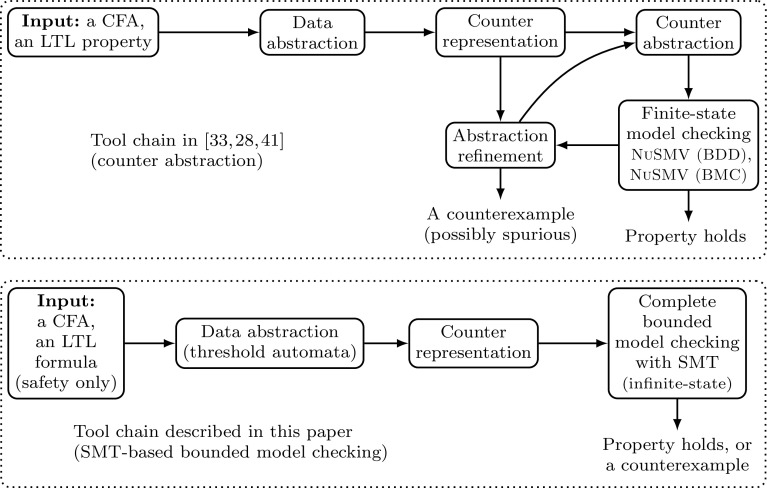
Tool chain with counter abstraction [27, 33, 41] on top, and with SMT-based bounded model checking on bottom

Figure 1 gives on top a diagram [40] that shows the technique based on counter abstraction. While this allowed us to automatically verify several FTDAs not verified before, there remained two bottlenecks for scalability to larger and more complex protocols: First, counter abstraction can lead to spurious counterexamples. As counters range over a finite abstract domain, the semantics of abstract increment and decrements on the counters introduce non-determinism. For instance, the value of a counter can remain unchanged after applying an increment. Intuitively, processes or messages can be “added” or “lost”, which results in that, e.g., in the abstract model the number of messages sent is smaller than the number of processes that have sent a message, which obviously is spurious behavior. Second, counter abstraction works well in practice only for processes with a few dozens of local states. It has been observed in [4] that counter abstraction does not scale to hundreds of local states. We had similar experience with counter abstraction in our experiments in [41]. We conjecture that this is partly due to the many different interleavings, which result in a large search space.

To address these bottlenecks, we make two crucial *contributions* in this paper:

1. To eliminate one of the two sources of spurious counterexamples, namely, the non-determinism added by abstract counters, we do bounded model checking using SMT solvers with linear integer arithmetic on the accelerated system, instead of SAT-based bounded model checking on the counter abstraction.
2. We reduce the search space dramatically: we introduce the notion of an *execution schema* that is defined as a sequence of local rules of the TA. By assigning to each rule of a schema an acceleration factor (possibly 0, which models that no process executes the rule), one obtains a run of the counter system. Hence, due to parameterization, each schema represents infinitely many runs. We show how to construct a set of schemas whose set of reachable states coincides with the set of reachable states of the accelerated counter system.

The resulting method is depicted at the bottom of Fig. 1. Our construction can be seen as an aggressive form of reduction, where each run has a similar run generated by a schema from the set. To show this, we capture the guards that are locked and unlocked in a *context*. Our key insight is that a bounded number of transitions changes the context in each run. For example, of all transitions increasing a variable *x*, at most one makes $x \ge n-t$ true, and at most one makes $x<t+1$ false (the parameters *n* and *t* are fixed in a run, and shared variables can only be increased). We fix those transitions that change the context, and apply the ideas of reduction to the subexecutions between these transitions.

Our experiments show that SMT solvers and schemas outperform SAT solvers and counter abstraction in parameterized verification of threshold-based FTDAs. We verified safety of FTDAs [10, 18, 29, 51, 56, 57] that have not been automatically verified before. In addition we achieved dramatic speedup and reduced memory footprint for FTDAs [9, 12, 58] which previously were verified in [41].

In this article we focus on parameterized reachability properties. Recently, we extended this approach to safety and liveness, for which we used the reachability technique of this article as a black box [37].

## Our approach at a glance

**Fig. 2:**
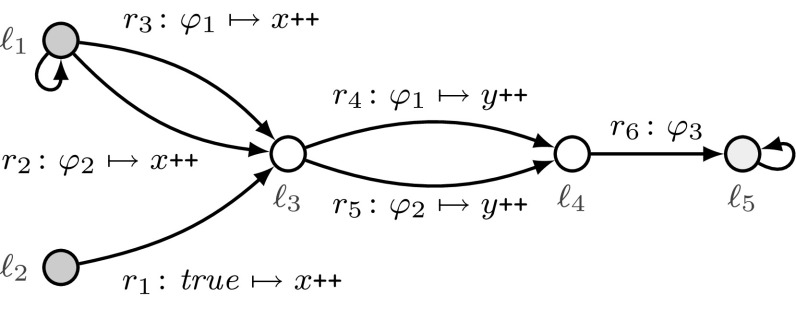
An example threshold automaton with threshold guards “$\varphi _1 :x \ge \lceil (n+t)/2 \rceil - f$”, “$\varphi _2 :y \ge (t + 1) - f$”, and “$\varphi _3 :y \ge (2t + 1) - f$”

For modeling threshold-based FTDAs, we use threshold automata that were introduced in [38, 41] and are discussed in more detail in [40]. We use Fig. 2 to describe our contributions in this section. The figure presents a threshold automaton TA over two shared variables *x* and *y* and parameters *n*, *t*, and *f*, which is inspired by the distributed asynchronous broadcast protocol from [9]. There, $n - f$ correct processes concurrently follow the control flow of TA, and *f* processes are Byzantine faulty. As is typical for FTDAs, the parameters must satisfy a resilience condition, e.g., $n > 3t \wedge t \ge f \ge 0$, that is, less than a third of the processes are faulty. The circles depict the local states $\ell _1, \dots , \ell _5$, two of them are the initial states $\ell _1$ and $\ell _2$. The edges depict the rules $r_1, \dots , r_6$ labeled with guarded commands $\varphi \mapsto \mathsf {act}$, where $\varphi$ is one of the threshold guards “$\varphi _1 :x \ge \lceil (n+t)/2 \rceil - f$”, “$\varphi _2 :y \ge (t + 1) - f$”, and “$\varphi _3 :y \ge (2t + 1) - f$”, and an action $\mathsf {act}$ increases the shared variables (*x* and *y*) by one, or zero (as in rule $r_6$).

We associate with every local state $\ell _i$ a non-negative counter ${\mathbf {\varvec{\kappa }}}[\ell _i]$ that represents the number of processes in $\ell _i$. Together with the values of *x*, *y*, *n*, *t*, and *f*, the values of the counters constitute a *configuration* of the system. In the initial configuration there are $n-f$ processes in initial states, i.e., ${\mathbf {\varvec{\kappa }}}[\ell _1] + {\mathbf {\varvec{\kappa }}}[\ell _2] = n - f$, and the other counters and the shared variables *x* and *y* are zero.

The rules define the transitions of the counter system. For example, according to the rule $r_2$, if in the current configuration the guard $y \ge t+1-f$ holds true and ${\mathbf {\varvec{\kappa }}}[\ell _1] \ge 5$, then five processes can instantaneously move out of the local state $\ell _1$ to the local state $\ell _3$, and increment *x* as prescribed by the action of $r_2$ (since the evaluation of the guard $y \ge t+1-f$ cannot change from true to false). This results in increasing *x* and ${\mathbf {\varvec{\kappa }}}[\ell _3]$ by five, and decreasing the counter ${\mathbf {\varvec{\kappa }}}[\ell _1]$ by five. When, as in our example, rule $r_2$ is conceptually executed by 5 processes, we denote this transition by $(r_2,5)$, where 5 is the acceleration factor. A sequence of transitions forms a *schedule*, e.g., $(r_1,2), (r_3,1), (r_1,1)$.

In this paper, we address a parameterized reachability problem, e.g., can at least one correct process reach the local state $\ell _5$, when $n-f$ correct processes start in the local state $\ell _1$? Or, in terms of counter systems, is a configuration with ${\mathbf {\varvec{\kappa }}}[\ell _5] \ne 0$ reachable from an initial configuration with ${\mathbf {\varvec{\kappa }}}[\ell _1] = n-f \wedge {\mathbf {\varvec{\kappa }}}[\ell _2] = 0$? As discussed in [41], acceleration does not affect reachability, and precise treatment of the resilience condition and threshold guards is crucial for solving this problem.

### Schemas

When applied to a configuration, a schedule generates a *path*, that is, an alternating sequence of configurations and transitions. As initially *x* and *y* are zero, threshold guards $\varphi _1$, $\varphi _2$, and $\varphi _3$ evaluate to false. As rules may increase variables, these guards may eventually become true. In our example we do not consider guards like $x<t+1$ that are initially true and become false, although we formally treat them in our technique. In fact, initially only $r_1$ is unlocked. Because $r_1$ increases *x*, it may unlock $\varphi _1$. Thus $r_4$ becomes unlocked. Rule $r_4$ increases *y* and thus repeated application of $r_4$ (by different processes) first unlocks $\varphi _2$ and then $\varphi _3$. We introduce a notion of a *context* that is the set of threshold guards that evaluate to true in a configuration. For our example we observe that each path goes through the following sequence of contexts $\{\}$, $\{\varphi _1\}$, $\{\varphi _1, \varphi _2\}$, and $\{\varphi _1, \varphi _2, \varphi _3\}$. In fact, the sequence of contexts in a path is always monotonic, as the shared variables can only be increased.

The conjunction of the guards in the context $\{\varphi _1, \varphi _2\}$ implies the guards of the rules $r_1, r_2, r_3, r_4, r_5$; we call these rules unlocked in the context. This motivates our definition of a *schema*: a sequence of contexts and rules. We give an example of a schema below, where inside the curly brackets we give the contexts, and fixed sequences of rules in between. (We discuss the underlined rules below.)2.1Given a schema, we can generate a schedule by attaching to each rule an acceleration factor, which can possibly be 0. For instance, if we attach non-zero factors to the underlined rules in *S*, and a zero factor to the other rules, we generate the following schedule $\tau '$ (we omit the transitions with 0 factors here).2.2It can easily be checked that $\tau '$ is generated by schema *S*, because the sequence of the underlined rules in *S* matches the sequence of rules appearing in $\tau '$.

In this paper, we show that the schedules generated by a few schemas —one per each monotonic sequence of contexts —span the set of all reachable configurations. To this end, we apply reduction and acceleration to relate arbitrary schedules to their representatives, which are generated by schemas.

### Reduction and acceleration

In this section we show what we mean by a schedule being “related” to its representative. Consider, e.g., the following schedule $\tau$ from the initial state $\sigma _0$ with $n=5$, $t=f=1$, ${\mathbf {\varvec{\kappa }}}[\ell _1] = 1$, and ${\mathbf {\varvec{\kappa }}}[\ell _2] = 3$:Observe that after $(r_1,1),(r_1,1)$, variable $x=2$, and thus $\varphi _1$ is true. Hence transition $t_1$ changes the context from $\{\}$ to $\{\varphi _1 \}$. Similarly $t_2$ and $t_3$ change the context. Context changing transitions are marked with curly brackets. Between them we have the subschedules $\tau _1, \dots , \tau _4$ ($\tau _3$ is empty) marked with square brackets.

To show that this schedule is captured by the schema (2.1), we apply partial order arguments —that is, a mover analysis [48] —regarding distributed computations: As the guards $\varphi _2$ and $\varphi _3$ evaluate to true in $\tau _4$, and $r_5$ precedes $r_6$ in the control flow of the TA, all transitions $(r_5,1)$ can be moved to the left in $\tau _4$. Similarly, $(r_1,1)$ can be moved to the left in $\tau _2$. The resulting schedule is applicable and leads to the same configuration as the original one. Further, we can accelerate the adjacent transitions with the same rule, e.g., the sequence $(r_5,1), (r_5,1)$ can be transformed into $(r_5,2)$. Thus, we transform subschedules $\tau _i$ into $\tau '_i$, and arrive at the schedule $\tau '$ from Eq. (2.2), which we call the representative schedule of $\tau$. As the representative schedule $\tau '$ is generated from the schema in (2.1), we say that the schema captures schedule $\tau$. (It also captures $\tau '$.) Importantly for reachability checking, if $\tau$ and $\tau '$ are applied to the same configuration, they end in the same configuration. These arguments are formalized in Sects. 5, 6 and 7.

### Encoding a schema in SMT

One of the key insights in this paper is that reachability checking via schemas can be encoded efficiently as SMT queries in linear integer arithmetic. In more detail, finite paths of counter systems can be expressed with inequalities over counters such as ${\mathbf {\varvec{\kappa }}}[\ell _2]$ and ${\mathbf {\varvec{\kappa }}}[\ell _3]$, shared variables such as *x* and *y*, parameters such as *n*, *t*, and *f*, and acceleration factors. Also, threshold guards and resilience conditions are expressions in linear integer arithmetic.

We give an example of reachability checking with SMT using the *simple* schema $\{\}\ r_1, r_1 \, \{\varphi _1\}$ which is contained in the schema *S* in Eq. (2.1). To obtain a complete encoding for *S*, one can similarly encode the other simple schemas and combine them.

To this end, we have to express constraints on three configurations $\sigma _0$, $\sigma _1$, and $\sigma _2$. For the initial configuration $\sigma _0$, we introduce integer variables: ${\mathbf {\varvec{\kappa }}}^0_1, \dots , {\mathbf {\varvec{\kappa }}}^0_5$ for local state counters, $x^0$ and $y^0$ for shared variables, and *n*, *t*, and *f* for parameters. As is written in Eq. (2.3), the configuration $\sigma _0$ should satisfy the initial constraints, and its context should be empty (that is, all guards evaluate to false):

2.3 $$\begin{aligned} {\mathbf {\varvec{\kappa }}}^0_1 + {\mathbf {\varvec{\kappa }}}^0_2= & {} n-f \wedge {\mathbf {\varvec{\kappa }}}^0_3 = {\mathbf {\varvec{\kappa }}}^0_4 = {\mathbf {\varvec{\kappa }}}^0_5 = 0 \wedge x^0 = y^0 = 0 \nonumber \\&\wedge n \ge 3t \wedge t \ge f \ge 0 \wedge (\lnot \varphi _1 \wedge \lnot \varphi _2 \wedge \lnot \varphi _3)[x^0/x,y^0/y] \end{aligned}$$

The configuration $\sigma _1$ is reached from $\sigma _0$ by applying a transition with the rule $r_1$ and an acceleration factor $\delta ^1$, and the configuration $\sigma _2$ is reached from $\sigma _1$ by applying a transition with the rule $r_1$ and an acceleration factor $\delta ^2$. Applying transition with the rule $r_1$ to $\sigma _0$ just means to increase both ${\mathbf {\varvec{\kappa }}}[\ell _3]$ and *x* by $\delta ^1$ and decrease ${\mathbf {\varvec{\kappa }}}[\ell _2]$ by $\delta ^1$. Hence, we introduce four fresh variables per transition and add the arithmetic operations. According to the schema, configuration $\sigma _2$ has the context $\{\varphi _2\}$. The following equations express these constraints^1^:

2.4 $$\begin{aligned} {\mathbf {\varvec{\kappa }}}^1_3= & {} {\mathbf {\varvec{\kappa }}}^0_3 + \delta ^1 \wedge {\mathbf {\varvec{\kappa }}}^1_2 = {\mathbf {\varvec{\kappa }}}^0_2 - \delta ^1 \wedge x^1 = x^0 + \delta ^1 \end{aligned}$$

2.5 $$\begin{aligned} {\mathbf {\varvec{\kappa }}}^2_3= & {} {\mathbf {\varvec{\kappa }}}^1_3 + \delta ^2 \wedge {\mathbf {\varvec{\kappa }}}^2_2 = {\mathbf {\varvec{\kappa }}}^1_2 - \delta ^2 \wedge x^2 = x^1 + \delta ^2 \nonumber \\&\quad \wedge (\varphi _1 \wedge \lnot \varphi _2 \wedge \lnot \varphi _3)[x^2/x,y^0/y] \end{aligned}$$

Finally, we express the reachability question for all paths generated by the simple schema $\{\}\ r_1 , r_1 \, \{\varphi _1\}$. Whether there is a configuration with ${\mathbf {\varvec{\kappa }}}[\ell _5] \ne 0$ reachable from an initial configuration with ${\mathbf {\varvec{\kappa }}}[\ell _1] = n -f$ and ${\mathbf {\varvec{\kappa }}}[\ell _2] = 0$ can then be encoded as:

2.6 $$\begin{aligned} {\mathbf {\varvec{\kappa }}}^0_1 = n -f \wedge {\mathbf {\varvec{\kappa }}}^0_2 = 0 \wedge {\mathbf {\varvec{\kappa }}}^0_5 \ne 0 \end{aligned}$$

Note that we check only ${\mathbf {\varvec{\kappa }}}^0_5$ against zero, as the local state $\ell _5$ is never updated by the rule $r_1$. It is easy to see that conjunction of Eqs. (2.3)–(2.6) does not have a solution, and thus all paths generated by the schema $\{\}\ r_1 , r_1 \, \{\varphi _1\}$ do not reach a configuration with ${\mathbf {\varvec{\kappa }}}[\ell _5] \ne 0$. By writing down constraints for the other three simple schemas in Eq. (2.1), we can check reachability for the paths generated by the whole schema as well. As discussed in Sect. 2.1, our results also imply reachability on all paths whose representatives are generated by the schema. More details on the SMT encoding can be found in Sect. 9.

## Parameterized counter systems

We recall the framework of [41] to the extent necessary, and extend it with the notion of a context in Sect. 3.2. A threshold automaton describes a process in a concurrent system, and is a tuple  defined below.

The finite set ${\mathcal L}$ contains the *local states*, and ${\mathcal I}\subseteq {\mathcal L}$ is the set of *initial states*. The finite set $\varGamma$ contains the *shared variables* that range over the natural numbers ${\mathbb N}_0$. The finite set $\Pi$ is a set of *parameter variables* that range over ${\mathbb N}_0$, and the *resilience condition* ${\textit{RC}}$ is a formula over parameter variables in linear integer arithmetic, e.g., $n>3t$. The set of *admissible parameters* is .

A key ingredient of threshold automata are threshold guards (or, just guards):

### Definition 3.1

A *threshold guard* is an inequality of one of the following two forms:

(R): $$x\ \ge \ a_0 + a_1 \cdot p_1 + \cdots + a_{{|\Pi |}} \cdot p_{|\Pi |}$$, or
(F): $$x\ <\ a_0 + a_1 \cdot p_1 + \cdots + a_{{|\Pi |}} \cdot p_{|\Pi |}$$,

where $x \in \varGamma$ is a shared variable, $a_0, \ldots , a_{|\Pi |}\in {\mathbb Z}$ are integer coefficients, and $p_1, \ldots , p_{|\Pi |}\in \Pi$ are parameters. We denote the set of all guards of the form (R) by $\varPhi ^{\mathrm {rise}}$, and the set of all guards of the form (F) by $\varPhi ^{\mathrm {fall}}$.

A rule defines a conditional transition between local states that may update the shared variables. Formally, a *rule* is a tuple $({ from }, { to }, \varphi ^{\mathrm {rise}}, \varphi ^{\mathrm {fall}}, \mathbf {u})$: the local states ${ from }$ and ${ to }$ are from ${\mathcal L}$. (Intuitively, they capture from which local state to which a process moves.) A rule is only executed if the conditions $\varphi ^{\mathrm {rise}}$ and $\varphi ^{\mathrm {fall}}$ evaluate to true. Condition $\varphi ^{\mathrm {rise}}$ is a conjunction of guards from $\varPhi ^{\mathrm {rise}}$, and $\varphi ^{\mathrm {fall}}$ is a conjunction of guards from $\varPhi ^{\mathrm {fall}}$ (cf. Definition 3.1). We denote the set of guards used in $\varphi ^{\mathrm {rise}}$ by $\mathsf {guard}(\varphi ^{\mathrm {rise}})$, and $\mathsf {guard}(\varphi ^{\mathrm {fall}})$ is the set of guards used in $\varphi ^{\mathrm {fall}}$.

Rules may increase shared variables using an update vector $\mathbf {u}\in {\mathbb N}_0^{|\varGamma |}$ that is added to the vector of shared variables. As $\mathbf {u}\in {\mathbb N}_0^{|\varGamma |}$, global variables can only be increased or left unchanged. As will be later formalized in Proposition 3.1, guards from $\varPhi ^{\mathrm {rise}}$ can only change from false to true (rise), and guards from $\varPhi ^{\mathrm {fall}}$ can change from true to false (fall). Finally, ${\mathcal R}$ is the finite set of rules. We use the dot notation to refer to components of rules, e.g., $r.{ from }$ or $r.\mathbf {u}$.

### Example 3.1

In Fig. 2, the rule $r_2:\varphi _2 \mapsto x{\texttt {++}}$ that describes a transition from $\ell _1$ to $\ell _3$, can formally be written as $(\ell _1, \ell _3, \varphi _2,\top , (1,0))$. Its intuitive meaning is as follows. If the guard $\varphi _2: y \ge (t+1)-f$ evaluates to true, a process can move from the local state $\ell _1$ to the local state $\ell _3$, and the global variable *x* is incremented, while *y* remains unchanged. We formalize the semantics as counter systems in Sect. 3.1.

### Definition 3.2

Given a threshold automaton $({\mathcal L}, {\mathcal I}, \varGamma , \Pi , {\mathcal R},{\textit{RC}})$, we define the *precedence relation* : for a pair of rules $r_1, r_2 \in {\mathcal R}$, it holds that $r_1 \prec _{{ P }}r_2$ if and only if $r_1.{ to }= r_2.{ from }$. We denote by $\prec ^+_{\scriptscriptstyle { P }}$ the transitive closure of $\prec _{{ P }}$. Further, we say that $r_1 \sim _{\scriptscriptstyle { P }}r_2$, if $r_1 \prec ^+_{\scriptscriptstyle { P }}r_2 \, \wedge \, r_2 \prec ^+_{\scriptscriptstyle { P }}r_1$, or $r_1 = r_2$.

### Assumption 3.3

We limit ourselves to threshold automata relevant for FTDAs, i.e., those where $r.\mathbf {u}= \mathbf {0}$ for all rules $r\in {\mathcal R}$ that satisfy $r \prec ^+_{\scriptscriptstyle { P }}r$. Such automata were called *canonical* in [41].

### Remark 3.1

We use threshold automata to model fault-tolerant distributed algorithms that count messages from distinct senders. These algorithms are based on an “idealistic” reliable communication assumption (no message loss); these assumptions are typically expected to be ensured by “lower level bookkeeping code”, e.g., communication protocols. As a result, the algorithms we consider here do not gain from sending the same message (that is, increasing a variable) inside a loop, so that we can focus on threshold automata that do not increase shared variables in loops.

### Example 3.2

**Fig. 3:**
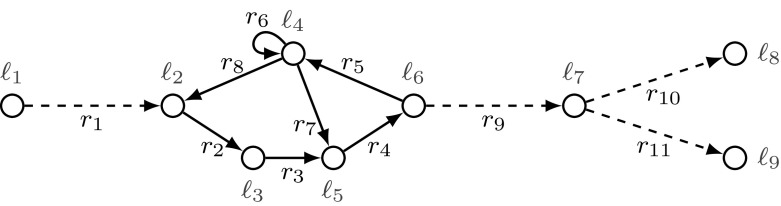
A threshold automaton $\textsf {TA}$ with local states ${\mathcal L}= \{\ell _i : 1\le i\le 9\}$ and rules ${\mathcal R}=\{ r_i : 1\le i\le 11\}$. The rules drawn with solid arrows $\{ r_2, \dots , r_8 \}$ constitute a single equivalence class, while all other rules are singleton equivalence classes

In the threshold automaton from Fig. 3 we have that $r_2\prec _{{ P }}r_3\prec _{{ P }}r_4 \prec _{{ P }}r_5 \prec _{{ P }}r_6 \prec _{{ P }}r_8 \prec _{{ P }}r_2$. Thus, we have that $r_2 \prec ^+_{\scriptscriptstyle { P }}r_2$. In our case this implies that $r_2.\mathbf {u}= \mathbf {0}$ by definition. Similarly we can conclude that $r_3.\mathbf {u}= r_4.\mathbf {u}= r_5.\mathbf {u}= r_6.\mathbf {u}= r_7.\mathbf {u}= r_8.\mathbf {u}= \mathbf {0}$.

*Looplets* The relation $\sim _{\scriptscriptstyle { P }}$ defines equivalence classes of rules. An equivalence class corresponds to a loop or to a single rule that is not part of a loop. Hence, we use the term looplet for one such equivalence class. For a given set of rules ${\mathcal R}$ let ${\mathcal R}/{\sim }$ be the set of equivalence classes defined by $\sim _{\scriptscriptstyle { P }}$. We denote by $[{r}]$ the equivalence class of rule *r*. For two classes $c_1$ and $c_2$ from ${\mathcal R}/{\sim }$ we write  iff there are two rules $r_1$ and $r_2$ in ${\mathcal R}$ satisfying $[{r_1}]=c_1$ and $[{r_2}]=c_2$ and $r_1 \prec ^+_{\scriptscriptstyle { P }}r_2$ and $r_1 \not \sim _{\scriptscriptstyle { P }}r_2$. As the relation  is a strict partial order, there are linear extensions of . Below, we fix an *arbitrary* of these linear extensions to sort transitions in a schedule: We denote by  a linear extension of .

### Example 3.3

Consider Fig. 3. The threshold automaton has five looplets: $c_1=\{r_1\}$, $c_2=\{r_2, \dots , r_8\}$, $c_3=\{r_9\}$, $c_4=\{r_{10}\}$, and $c_5=\{r_{11}\}$. From $r_9 \prec _{{ P }}r_{10}$, it follows that , and from $r_4\prec ^+_{\scriptscriptstyle { P }}r_{10}$, it follows that . We can pick two linear extensions of , denoted by  and . We have , and . In this paper we always fix one linear extension.

**Fig. 4:**
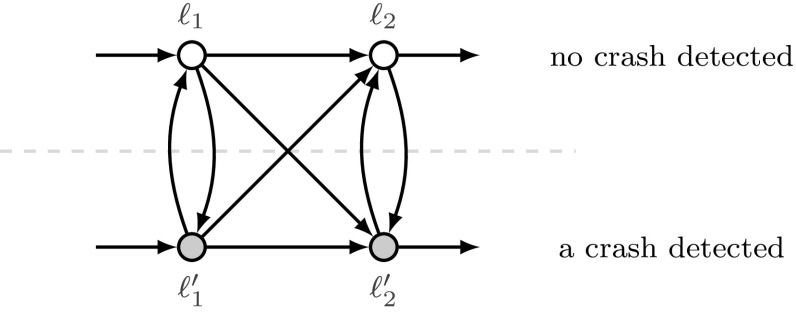
A typical structure found in threshold automata that model fault-tolerant algorithms with a failure detector [12]. The gray circles depict those local states, where the failure detector reports a crash. The local states $\ell _i$ and $\ell '_i$ differ only in the output of the failure detector. As the failure detector output changes non-deterministically, the threshold automaton contains loops of size two

### Remark 3.2

It may seem natural to collapse such loops into singleton local states. In our case studies, e.g, [29], non-trivial loops are used to express non-deterministic choice due to failure detectors [12], as shown in Fig. 4. Importantly, some local states inside the loops appear in the specifications. Thus, one would have to use arguments from distributed computing to characterize when collapsing states is sound. In this paper, we present a technique that deals with the loops without need for additional modelling arguments.

### Counter systems

We use a function $N:\mathbf {P}_{RC}\rightarrow {\mathbb N}_0$ to capture the number of processes for each combination of parameters. As we use SMT, we assume that $N$ can be expressed in linear integer arithmetic. For instance, if only correct processes are explictly modeled we typically have $N(n,t,f) = n-f$, and the respective SMT expression is $n - f$. Given $N$, a threshold automaton $\textsf {TA}$, and admissible parameter values $\mathbf {p}\in \mathbf {P}_{RC}$, we define a counter system as a transition system $(\varSigma ,I,R)$. It consists of the set of configurations $\varSigma$, which contain evaluations of the counters and variables, the set of initial configurations $I$, and the transition relation $R$:

*Configurations* $\varSigma$ *and* $I$ A configuration $\sigma =({\mathbf {\varvec{\kappa }}},\mathbf {g},\mathbf {p})$ consists of a vector of *counter values* $\sigma .{\mathbf {\varvec{\kappa }}}\in {\mathbb N}_0^{|{\mathcal L}|}$ (for simplicity we use the convention that ${\mathcal L}= \{1, \ldots , |{\mathcal L}| \}$) a vector of *shared variable values* $\sigma .\mathbf {g}\in {\mathbb N}_0^{|\varGamma |}$, and a vector of *parameter values* $\sigma .\mathbf {p}= \mathbf {p}$. The set $\varSigma$ is the set of all configurations. The set of initial configurations $I$ contains the configurations that satisfy $\sigma .\mathbf {g}= \mathbf {0}$, $\sum _{i \in {\mathcal I}} \sigma .{\mathbf {\varvec{\kappa }}}[i] = N(\mathbf {p})$, and $\sum _{i \not \in {\mathcal I}} \sigma .{\mathbf {\varvec{\kappa }}}[i] = 0$. This means that in every initial configuration all global variables have zero values, and all $N(\mathbf {p})$ modeled processes are located only in the initial local states.

#### Example 3.4

Consider the threshold automaton from Fig. 2 with the initial states $\ell _1$ and $\ell _2$. Let us consider a system of five processes, one of them being Byzantine faulty. Thus, $n=5$, $t=f=1$, and we explicitely model $N(5,1,1)=n-f=4$ correct processes. One of the initial configurations is $\sigma =({\mathbf {\varvec{\kappa }}},\mathbf {g},\mathbf {p})$, where $\sigma .{\mathbf {\varvec{\kappa }}}=(1,3,0,0,0)$, $\sigma .\mathbf {g}=(0,0)$, and $\sigma .\mathbf {p}= (5,1,1)$. In other words, there is one process in $\ell _1$, three processes in $\ell _2$, and global variables are initially $x=y=0$. Note that $\sum _{i\in {\mathcal I}} \sigma .{\mathbf {\varvec{\kappa }}}[i] = {\mathbf {\varvec{\kappa }}}[\ell _1] + {\mathbf {\varvec{\kappa }}}[\ell _2] = 1+3=4=N(5,1,1)$.

*Transition relation* $R$ A *transition* is a pair $t=({ rule },{ factor })$ of a rule of the $\textsf {TA}$ and a non-negative integer called the *acceleration factor*, or just factor for short. (As already discussed in Sect. 2.1, we will use the zero factors when generating schedules from schemas.) For a transition $t=({ rule },{ factor })$ we refer by $t.\mathbf {u}$ to ${ rule }.\mathbf {u}$, and by $t.\varphi ^{\mathrm {fall}}$ to ${ rule }.\varphi ^{\mathrm {fall}}$, etc. We say a transition *t* is *unlocked* in configuration $\sigma$ if $(\sigma .{\mathbf {\varvec{\kappa }}},\sigma .\mathbf {g}+ k\cdot t.\mathbf {u}, \sigma .\mathbf {p}) \models t.\varphi ^{\mathrm {rise}}\wedge t.\varphi ^{\mathrm {fall}}$, for $k \in \{0, \ldots , t.{ factor }- 1 \}$. Note that here we use a notation that a configuration satisfies a formula, which is considered true if and only if the formula becomes true when all free variables of the formulas are evaluated as in the configuration.

We say that transition *t* is *applicable (or enabled)* in configuration $\sigma$, if it is unlocked, and $\sigma .{\mathbf {\varvec{\kappa }}}[t.{ from }] \ge t.{ factor }$. (As all counters are non-negative, a transition with the zero ${ factor }$ is always applicable to all configurations provided that the guards are unlocked.) We say that $\sigma '$ is the result of applying the enabled transition *t* to $\sigma$, and write $\sigma ' = t(\sigma )$, if

- $$\sigma '.\mathbf {g}= \sigma .\mathbf {g}+ t.{ factor }\cdot t.\mathbf {u}$$ and $$\sigma '.\mathbf {p}= \sigma .\mathbf {p}$$
- if $$t.{ from }\ne t.{ to }$$ then
  - $$\sigma '.{\mathbf {\varvec{\kappa }}}[t.{ from }]=\sigma .{\mathbf {\varvec{\kappa }}}[t.{ from }] - t.{ factor }$$,
  - $$\sigma '.{\mathbf {\varvec{\kappa }}}[t.{ to }]=\sigma .{\mathbf {\varvec{\kappa }}}[t.{ to }] + t.{ factor }$$, and
  - $\forall \ell \in {\mathcal L}{\setminus } \{t.{ from }, t.{ to }\}$ it holds that $\sigma '.{\mathbf {\varvec{\kappa }}}[\ell ]=\sigma .{\mathbf {\varvec{\kappa }}}[\ell ]$
- if $$t.{ from }= t.{ to }$$ then $$\sigma '.{\mathbf {\varvec{\kappa }}}= \sigma .{\mathbf {\varvec{\kappa }}}$$

The transition relation $R\subseteq \varSigma \times \varSigma$ of the counter system is defined as follows: $(\sigma , \sigma ') \in R$ iff there is a rule $r\in {\mathcal R}$ and a factor $k\in {\mathbb N}_0$ such that $\sigma ' = t(\sigma )$ for $t=(r,k)$. Updates do not decrease the values of shared variables, and thus the following proposition was introduced in [41]:

#### Proposition 3.1

[41] For all configurations $\sigma$, all rules *r*, and all transitions *t* applicable to $\sigma$, the following holds:

1.  If $\sigma \models r.\varphi ^{\mathrm {rise}}$ then $t(\sigma ) \models r.\varphi ^{\mathrm {rise}}$             3.  If $\sigma \not \models r.\varphi ^{\mathrm {fall}}$ then $t(\sigma ) \not \models r.\varphi ^{\mathrm {fall}}$

2.  If $t(\sigma ) \not \models r.\varphi ^{\mathrm {rise}}$ then $\sigma \not \models r.\varphi ^{\mathrm {rise}}$             4.  If $t(\sigma ) \models r.\varphi ^{\mathrm {fall}}$ then $\sigma \models r.\varphi ^{\mathrm {fall}}$

*Schedules and paths* A *schedule* is a (finite) sequence of transitions. For a schedule $\tau$ and an index $i: 1 \le i \le |\tau |$, by $\tau [i]$ we denote the *i*th transition of $\tau$, and by $\tau ^i$ we denote the prefix $\tau [1], \ldots , \tau [i]$ of $\tau$. A schedule $\tau = t_1, \ldots , t_m$ is *applicable* to configuration $\sigma _0$, if there is a sequence of configurations $\sigma _1,\ldots , \sigma _m$ with $\sigma _i = t_{i} (\sigma _{i-1})$ for $1 \le i \le m$. If there is a $t_i.{ factor }>1$, then a schedule is *accelerated*.

By $\tau \cdot \tau '$ we denote the concatenation of two schedules $\tau$ and $\tau '$. A sequence $\sigma _0, t_1, \sigma _1, \ldots , \sigma _{k-1}, t_k, \sigma _k$ of alternating configurations and transitions is called a (finite) *path*, if transition $t_i$ is enabled in $\sigma _{i-1}$ and $\sigma _i = t_i(\sigma _{i-1})$, for $1 \le i \le k$. For a configuration $\sigma _0$ and a schedule $\tau$ applicable to $\sigma _0$, by $\textsf {path}(\sigma _0, \tau )$ we denote the path $\sigma _0, t_1, \ldots , t_{|\tau |}, \sigma _{|\tau |}$ with $t_i = \tau [i]$ and $\sigma _i = t_i(\sigma _{i-1})$, for $1 \le i \le |\tau |$.

### Contexts and slices

The evaluation of the guards in the sets $\varPhi ^{\mathrm {rise}}$ and $\varPhi ^{\mathrm {fall}}$ in a configuration solely defines whether certain transitions are unlocked (but not necessarily enabled). From Proposition 3.1, one can see that after a transition has been applied, more guards from $\varPhi ^{\mathrm {rise}}$ may get unlocked and more guards from $\varPhi ^{\mathrm {fall}}$ may get locked. In other words, more guards from $\varPhi ^{\mathrm {rise}}$ may evaluate to true and more guards from $\varPhi ^{\mathrm {fall}}$ may evaluate to false. To capture this intuition, we define:

#### Definition 3.4

A *context* $\varOmega$ is a pair $(\varOmega ^\mathrm {rise}, \varOmega ^{\mathrm {fall}})$ with $\varOmega ^\mathrm {rise}\subseteq \varPhi ^{\mathrm {rise}}$ and $\varOmega ^{\mathrm {fall}}\subseteq \varPhi ^{\mathrm {fall}}$. We denote by $|\varOmega | = |\varOmega ^\mathrm {rise}| + |\varOmega ^{\mathrm {fall}}|$.

For two contexts $(\varOmega ^\mathrm {rise}_1, \varOmega ^{\mathrm {fall}}_1)$ and $(\varOmega ^\mathrm {rise}_2, \varOmega ^{\mathrm {fall}}_2)$, we define that $(\varOmega ^\mathrm {rise}_1, \varOmega ^{\mathrm {fall}}_1) \sqsubset (\varOmega ^\mathrm {rise}_2, \varOmega ^{\mathrm {fall}}_2)$ if and only if $\varOmega ^\mathrm {rise}_1 \cup \varOmega ^{\mathrm {fall}}_1 \subset \varOmega ^\mathrm {rise}_2 \cup \varOmega ^{\mathrm {fall}}_2$. Then, a sequence of contexts $\varOmega _1, \ldots , \varOmega _m$ is *monotonically increasing*, if $\varOmega _i \sqsubset \varOmega _{i+1}$, for $1 \le i < m$. Further, a monotonically increasing sequence of contexts $\varOmega _1, \ldots , \varOmega _m$ is *maximal*, if $\varOmega _1 = (\emptyset , \emptyset )$ and $\varOmega _m = (\varPhi ^{\mathrm {rise}}, \varPhi ^{\mathrm {fall}})$ and $|\varOmega _{i+1}| = |\varOmega _i| + 1$, for $1 \le i < m$. We obtain:

#### Proposition 3.2

Every maximal monotonically increasing sequence of contexts is of length $|\varPhi ^{\mathrm {rise}}| + |\varPhi ^{\mathrm {fall}}| + 1$. There are at most $(|\varPhi ^{\mathrm {rise}}| + |\varPhi ^{\mathrm {fall}}|)!\,$ such sequences.

#### Example 3.5

For the example in Fig. 2, we have $\varPhi ^{\mathrm {rise}}= \{\varphi _1, \varphi _2, \varphi _3\}$, and $\varPhi ^{\mathrm {fall}}= \emptyset$. Thus, there are $(|\varPhi ^{\mathrm {rise}}| + |\varPhi ^{\mathrm {fall}}|)!=6$ maximal monotonically increasing sequences of contexts. Two of them are $(\emptyset ,\emptyset )\sqsubset (\{\varphi _1\},\emptyset ) \sqsubset (\{\varphi _1,\varphi _2\},\emptyset )\sqsubset ( \{\varphi _1, \varphi _2, \varphi _3\},\emptyset )$ and $(\emptyset ,\emptyset )\sqsubset (\{\varphi _3\},\emptyset )\sqsubset (\{\varphi _1,\varphi _3\},\emptyset ) \sqsubset (\{\varphi _1,\varphi _2, \varphi _3\}, \emptyset )$. All of them have length $|\varPhi ^{\mathrm {rise}}| + |\varPhi ^{\mathrm {fall}}| + 1=4$.

To every configuration $\sigma$, we attach the context consisting of all guards in $\varPhi ^{\mathrm {rise}}$ that evaluate to true in $\sigma$, and all guards in $\varPhi ^{\mathrm {fall}}$ that evaluate to *false* in $\sigma$:

#### Definition 3.5

Given a threshold automaton, we define its *configuration context* as a function $\omega : \varSigma \rightarrow 2^{\varPhi ^{\mathrm {rise}}} \times 2^{\varPhi ^{\mathrm {fall}}}$ that for each configuration $\sigma \in \varSigma$ gives a context $(\varOmega ^\mathrm {rise},\varOmega ^{\mathrm {fall}})$ with $\varOmega ^\mathrm {rise}= \{ \varphi \in \varPhi ^{\mathrm {rise}}:\sigma \models \varphi \}$ and $\varOmega ^{\mathrm {fall}}= \{ \varphi \in \varPhi ^{\mathrm {fall}}:\sigma \not \models \varphi \}$.

The following monotonicity result is a direct consequence of Proposition 3.1.

#### Proposition 3.3

If a transition *t* is enabled in a configuration $\sigma$, then either $\omega (\sigma ) \sqsubset \omega (t(\sigma ))$, or $\omega (\sigma ) = \omega (t(\sigma ))$.

#### Definition 3.6

A schedule $\tau$ is *steady* for a configuration $\sigma$, if for every prefix $\tau '$ of $\tau$, the context does not change, i.e., $\omega (\tau '(\sigma )) = \omega (\sigma )$.

#### Proposition 3.4

A schedule $\tau$ is steady for a configuration $\sigma$ if and only if $\omega (\sigma ) = \omega (\tau (\sigma ))$.

In the following definition, we associate a sequence of contexts with a path:

#### Definition 3.7

Given a configuration $\sigma$ and a schedule $\tau$ applicable to $\sigma$, we say that $\textsf {path}(\sigma , \tau )$ *is consistent with* a sequence of contexts $\varOmega _1, \ldots , \varOmega _m$, if there exist indices $n_0,\ldots ,n_{m}$, with $0=n_0\le n_1\le \ldots \le n_m=|\tau |+1$, such that for every *k*, $1 \le k \le m$, and every *i* with $n_{k-1}\le i < n_k$, it holds that $\omega (\tau ^i(\sigma )) = \varOmega _k$.

Every path is consistent with a uniquely defined maximal monotonically increasing sequence of contexts. (Some of the indices $n_0,\dots ,n_m$ in Definition 3.7 may be equal.) In Sect. 4, we use such sequences of contexts to construct a schema recognizing many paths that are consistent with the same sequence of contexts.

A context defines which rules of the $\textsf {TA}$ are unlocked. A schedule that is steady for a configuration visits only one context, and thus we can statically remove $\textsf {TA}$’s rules that are locked in the context:

#### Definition 3.8

Given a threshold automaton $\textsf {TA}=({\mathcal L}, {\mathcal I}, \varGamma , \Pi , {\mathcal R},{\textit{RC}})$ and a context $\varOmega$, we define the *slice* of $\textsf {TA}$ with context $\varOmega =(\varOmega ^\mathrm {rise},\varOmega ^{\mathrm {fall}})$ as a threshold automaton ${\textsf {TA}}|_{\varOmega }=({\mathcal L}, {\mathcal I}, \varGamma , \Pi , {{\mathcal R}}|_{\varOmega }, {\textit{RC}})$, where a rule $r\in {\mathcal R}$ belongs to ${{\mathcal R}}|_{\varOmega }$ if and only if $\big (\bigwedge _{\varphi \in \varOmega ^\mathrm {rise}} \varphi \big ) \rightarrow r.\varphi ^{\mathrm {rise}}$ and $\big (\bigwedge _{\psi \in \varPhi ^{\mathrm {fall}}{\setminus } \varOmega ^{\mathrm {fall}}} \psi \big ) \rightarrow r.\varphi ^{\mathrm {fall}}$.

**Fig. 5:**
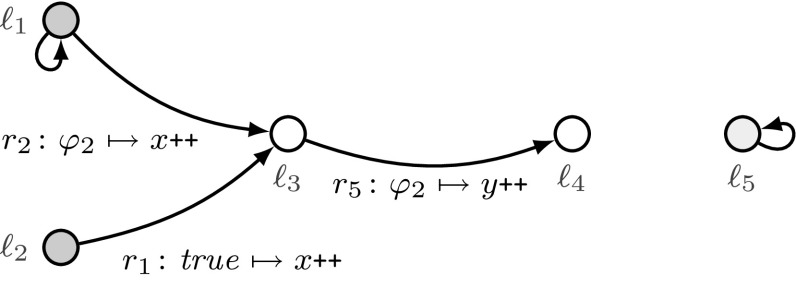
The slice of the $\textsf {TA}$ in Fig. 2 that is constructed for the context $(\{\varphi _2\}, \emptyset )$

In other words, ${{\mathcal R}}|_{\varOmega }$ contains those and only those rules *r* with guards that evaluate to true in all configurations $\sigma$ with $\omega (\sigma )=\varOmega$. These are exactly the guards from $\varOmega ^\mathrm {rise}\cup (\varPhi ^{\mathrm {fall}}{\setminus }\varOmega ^{\mathrm {fall}})$. When $\omega (\sigma )=\varOmega$, then all guards from $\varOmega ^\mathrm {rise}$ evaluate to true, and then $r.\varphi ^{\mathrm {rise}}$ must also be true. As $\varOmega ^{\mathrm {fall}}$ contains those guards from $\varPhi ^{\mathrm {fall}}$ that evaluate to false in $\sigma$, then all other guards from $\varPhi ^{\mathrm {fall}}$ must evaluate to true, and then $r.\varphi ^{\mathrm {fall}}$ must be true too. Figure 5 shows an example of a slice.

### Model checking problem: parameterized reachability

Given a threshold automaton $\textsf {TA}$, a *state property* *B* is a Boolean combination of formulas that have the form $\bigwedge _{i \in Y} {\mathbf {\varvec{\kappa }}}[i] = 0$, for some $Y \subseteq {\mathcal L}$. The *parameterized reachability* problem is to decide whether there are parameter values $\mathbf {p}\in \mathbf {P}_{RC}$, an initial configuration $\sigma _0 \in I$, with $\sigma _0.\mathbf {p}= \mathbf {p}$, and a schedule $\tau$, such that $\tau$ is applicable to $\sigma _0$, and property *B* holds in the final state: $\tau (\sigma _0) \models B$.

## Main result: a complete set of schemas

To address parameterized reachability, we introduce schemas, i.e., alternating sequences of contexts and rule sequences. A schema serves as a pattern for a set of paths, and is used to efficiently encode parameterized reachability in SMT. As parameters give rise to infinitely many initial states, a schema captures an *infinite* set of paths. We show how to construct a *finite* set of schemas $\mathcal{S}$ with the following property: for each schedule $\tau$ and each configuration $\sigma$ there is a representative schedule $s(\tau )$ such that: (1) applying $s(\tau )$ to $\sigma$ results in $\tau (\sigma )$, and (2) $\textsf {path}(\sigma , s(\tau ))$ is generated by a schema from $\mathcal{S}$.

### Definition 4.1

A schema is a sequence $\varOmega _0, \rho _1, \varOmega _1, \dots , \rho _m, \varOmega _m$ of alternating contexts and rule sequences. We often write $\{\varOmega _0\} \rho _1 \{\varOmega _1\} \dots \{\varOmega _{m-1}\} \rho _m \{\varOmega _m\}$ for a schema. A schema with two contexts is called simple.

Given two schemas $S_1 =\varOmega _0, \rho _1, \dots , \rho _k, \varOmega _k$ and $S_2 = \varOmega '_0, \rho '_1, \dots , \rho '_m, \varOmega '_m$ with $\varOmega _k = \varOmega '_0$, we define their *composition* $S_1\,\circ \,S_2$ to be the schema that is obtained by concatenation of the two sequences: $\varOmega _0, \rho _1, \dots , \rho _k, \varOmega '_0, \rho '_1, \dots , \rho '_m, \varOmega '_m$.

### Definition 4.2

Given a configuration $\sigma$ and a schedule $\tau$ applicable to $\sigma$, we say that $\textsf {path}(\sigma , \tau )$ is generated by a simple schema $\{\varOmega \}\,\rho \,\{\varOmega '\}$, if the following hold:

- For $\rho = r_1, \dots , r_k$ there is a monotonically increasing sequence of indices $i(1), \dots , i(m)$, i.e., $1 \le i(1)< \cdots < i(m) \le k$, and there are factors $f_1, \dots , f_m \ge 0$, so that schedule $(r_{i(1)}, f_1), \dots , (r_{i(m)}, f_m) = \tau$.
- The first and the last states match the contexts: $\omega (\sigma ) = \varOmega$ and $\omega (\tau (\sigma )) = \varOmega '$.

In general, we say that $\textsf {path}(\sigma , \tau )$ is generated by a schema *S*, if $S = S_1\, \circ \dots \circ \, S_k$ for simple schemas $S_1, \dots , S_k$ and $\tau = \tau _1 \cdots \tau _k$ such that each $\textsf {path}(\pi _i(\sigma ), \tau _i)$ is generated by the simple schema $S_i$, for $\pi _i = \tau _1 \cdots \tau _{i-1}$ and $1 \le i \le k$.

### Remark 4.1

Definition 4.2 allows schemas to generate paths that have transitions with zero acceleration factors. Applying a transition with a zero factor to a configuration $\sigma$ results in the same configuration $\sigma$, which corresponds to a stuttering step. This does not affect reachability. In the following, we will apply Definition 4.2 to representative paths that may have transitions with zero factors.

### Example 4.1

Let us go back to the example of a schema *S* and a schedule $\tau '$ introduced in Eqs. (2.1) and (2.2) in Sect. 2.1. It is easy to see that schema *S* can be decomposed into four simple schemas $S_1 \circ \dots \circ S_4$, e.g., $S_1 = \{\}\ r_1, r_1\ \{\varphi _1\}$ and $S_2=\{\varphi _1\}\ r_1, r_3, r_4, r_4\ \{\varphi _1, \varphi _2\}$. Consider an initial state $\sigma _0$ with $n=5$, $t=f=1$, $x=y=0$, ${\mathbf {\varvec{\kappa }}}[\ell _1] = 1$, ${\mathbf {\varvec{\kappa }}}[\ell _2] = 3$, and ${\mathbf {\varvec{\kappa }}}[\ell _i]=0$ for $i \in \{3, 4, 5\}$. To ensure that $\textsf {path}(\sigma _0, \tau ')$ is generated by schema *S*, one has to check Definition 4.2 for schemas $S_1, \dots , S_4$ and schedules $(\tau '_1 \cdot t_1)$, $(\tau '_2 \cdot t_2)$, $(\tau '_3 \cdot t_3)$, and $\tau '_4$, respectively. For instance, $\textsf {path}(\sigma _0, \tau '_1 \cdot t_1)$ is generated by $S_1$. Indeed, take the sequence of indices 1 and 2 and the sequence of acceleration factors 1 and 1. The path $\textsf {path}(\sigma _0, \tau '_1 \cdot t_1)$ ends in the configuration $\sigma _1$ that differs from $\sigma _0$ in that ${\mathbf {\varvec{\kappa }}}[\ell _2]=1$, ${\mathbf {\varvec{\kappa }}}[\ell _3]=2$, and $x=2$. The contexts $\omega (\sigma _0) = (\{\}, \{\})$ and $\omega (\sigma _1)=(\{\varphi _1\}, \{\})$ match the contexts of schema $S_1$, as required by Definition 4.2.

Similarly, $\textsf {path}(\sigma _1, \tau '_2 \cdot t_2)$ is generated by schema $S_2$. To see that, compare the contexts and use the index sequence 1, 2, 4, and acceleration factors 1.

The *language of a schema* *S* —denoted with $\mathcal{L}(S)$ —is the set of all paths generated by *S*. For a set of configurations $C \subseteq \varSigma$ and a set of schemas $\mathcal{S}$, we define the set $\textsf {Reach}(C, \mathcal{S})$ to contain all configurations reachable from *C* via the paths generated by the schemas from $\mathcal{S}$, i.e., $\textsf {Reach}(C, \mathcal{S}) = \{ \tau (\sigma ) \mid \sigma \in C,\ \exists S \in \mathcal{S}.\ \textsf {path}(\sigma , \tau ) \in \mathcal{L}(S) \}$. We say that a set $\mathcal{S}$ of schemas is *complete*, if for every set of configurations $C\subseteq \varSigma$ it is the case that the set of all states reachable from *C* via the paths generated by the schemas from $\mathcal{S}$, is exactly the set of all possible states reachable from *C*. Formally, $\forall C \subseteq \varSigma .\; \{ \tau (\sigma ) \mid \sigma \in C,\ \tau \text{ is } \text{ applicable } \text{ to } \sigma \} = \textsf {Reach}(C, \mathcal{S})$.

In [41], a quantity ${\mathcal C}$ has been introduced that depends on the number of conditions in a TA. It has been shown that for every configuration $\sigma$ and every schedule $\tau$ applicable to $\sigma$, there is a schedule $\tau '$ of length at most $d=|{\mathcal R}| \cdot ({\mathcal C}+ 1) + {\mathcal C}$ that is also applicable to $\sigma$ and results in $\tau (\sigma )$ [41, Thm. 8]. Hence, by enumerating all sequences of rules of length up to *d*, one can construct a complete set of schemas:

### Theorem 4.1

For a threshold automaton, there is a complete schema set $\mathcal{S}_d$ of cardinality $|{\mathcal R}|^{|{\mathcal R}| \cdot ({\mathcal C}+ 1) + {\mathcal C}}$.

Although the set $\mathcal{S}_d$ is finite, enumerating all its elements is impractical. We show that there is a complete set of schemas whose cardinality solely depends on the number of guards that syntactically occur in the TA. These numbers $|\varPhi ^{\mathrm {rise}}|$ and $|\varPhi ^{\mathrm {fall}}|$ are in practice much smaller than the number of rules $|{\mathcal R}|$:

### Theorem 4.2

For all threshold automata, there exists a complete schema set of cardinality at most $(|\varPhi ^{\mathrm {rise}}| + |\varPhi ^{\mathrm {fall}}|)!$. In this set, the length of each schema does not exceed $(3 \cdot |\varPhi ^{\mathrm {rise}}\cup \varPhi ^{\mathrm {fall}}| + 2) \cdot |{\mathcal R}|$.

In the following sections we prove the ingredients of the following argument for the theorem: construct the set *Z* of all maximal monotonically increasing sequences of contexts. From Proposition 3.2, we know that there are at most $(|\varPhi ^{\mathrm {rise}}| +|\varPhi ^{\mathrm {fall}}|)!$ maximal monotonically increasing sequences of contexts. Therefore, $|Z| \le (|\varPhi ^{\mathrm {rise}}| + |\varPhi ^{\mathrm {fall}}|)!$. Then, for each sequence $z \in Z$, we do the following:

1. We show that for each configuration $\sigma$ and each schedule $\tau$ applicable to $\sigma$ and consistent with the sequence *z*, there is a schedule $s(\tau )$ that has a specific structure, and is also applicable to $\sigma$. We call $s(\tau )$ the representative of $\tau$. We introduce and formally define this specific structure of representative schedules in Sects. 5, 6 and 7. We prove existence and properties of the representative schedule in Theorem 7.1 (Sect. 7). Before that we consider special cases: when all rules of a schedule belong to the same looplet (Theorem 5.1, Sect. 5), and when a schedule is steady (Theorem 6.1, Sect. 6).
2. Next we construct a schema (for the sequence *z*) and show that it generates all paths of all schedules $s(\tau )$ found in (1). The length of the schema is at most $(3 \cdot (|\varPhi ^{\mathrm {rise}}| + |\varPhi ^{\mathrm {fall}}|) + 2) \cdot |{\mathcal R}|$. This is shown in Theorem 7.2 (Sect. 7).

Theorem 4.2 follows from the above theorems, which we prove in the following.

### Remark 4.2

Let us stress the difference between [41] and this work. From [41], it follows that in order to check correctness of a $\textsf {TA}$ it is sufficient to check only the schedules of bounded length $d(\textsf {TA})$. The bound $d(\textsf {TA})$ does not depend on the parameters, and can be computed for each $\textsf {TA}$. The proofs in [41] demonstrate that every schedule longer than $d(\textsf {TA})$ can be transformed into an “equivalent” representative schedule, whose length is bounded by $d(\textsf {TA})$. Consequently, one can treat every schedule of length up to $d(\textsf {TA})$ as its own representative schedule. Similar reasoning does not apply to the schemas constructed in this paper: (i) we construct a complete set of schemas, whose cardinality is substantially smaller than $|\mathcal{S}_d|$, and (ii) the schemas constructed in this paper can be twice as long as the schemas in $\mathcal{S}_d$.

As discussed in Remark 3.2, the looplets in our case studies are typically either singleton looplets or looplets of size two. In fact, most of our benchmarks have singleton looplets only, and thus their threshold automata can be reduced to directed acyclic graphs. The theoretical constructs of Sect. 5.2 are presented for the more general case of looplets of any size. For most of the benchmarks —the ones not using failure detectors —we need only the simple construction laid out in Sect. 5.1.

## Case I: one context and one looplet

We show that for each schedule that uses only the rules from a fixed looplet and does not change its context, there exists a representative schedule of bounded length that reaches the same final state. The goal is to construct a single schema per looplet. The technical challenge is that this *single* schema must generate representative schedules for *all* possible schedules, where, intuitively, processes may move arbitrarily between all local states in the looplet. As a consequence, the rules that appear in the representative schedules can differ from the rules that appear in the arbitrary schedules visiting a looplet.

We fix a threshold automaton, a context $\varOmega$, a configuration $\sigma$ with $\omega (\sigma ) = \varOmega$, a looplet *c*, and a schedule $\tau$ applicable to $\sigma$ and using only rules from *c*. We then construct the representative schedule $\mathsf {crep}^{\varOmega }_{c}[\sigma , \tau ]$ and the schema $\mathsf {cschema}^{\varOmega }_{c}$.

The technical details of the construction of $\mathsf {crep}^{\varOmega }_{c}[\sigma , \tau ]$ for the case when $|c|=1$ is given in Sect. 5.1, and for the case when $|c|>1$ in Sect. 5.2. We show in Sect. 5.3 that these constructions give us a schedule that has the desired properties: it reaches the same final state as the given schedule $\tau$, and its length does not exceed $2 \cdot |c|$.

Note that in [41], the length of the representative schedule was bounded by |*c*|. However, all representative schedules of a looplet in this section can be generated by a single looplet schema.

### Singleton looplet

Let us consider the case of the looplet *c* containing only one transition, that is, $|c|=1$. There is a trivial representative schedule of a single transition:

#### Lemma 5.1

Given a threshold automaton, a configuration $\sigma$, and a schedule $\tau = (r, f_1)$, ..., $(r, f_m)$ applicable to $\sigma$, one of the two schedules is also applicable to $\sigma$ and results in $\tau (\sigma )$: schedule $(r, f_1 + \cdots + f_m)$, or schedule (*r*, 0).

#### Proof

We distinguish two cases:

*Case* ${{r.to} = {r.from}}$ Then, $r.\mathbf {u}= \mathbf {0}$, and $\tau ^k(\sigma ) = \sigma$ for $0 \le k \le |\tau |$. Consequently, the schedule (*r*, 0) is applicable to $\sigma$, and it results in $\tau (\sigma )=\sigma$.

*Case* ${r.to} \ne {r.from}$ We prove by induction on the length $k: 1 \le k \le m$ of a prefix of $\tau$, that the following constraints hold for all *k*:

5.1 $$\begin{aligned}&(\tau ^k(\sigma )).{\mathbf {\varvec{\kappa }}}[r.{ from }] = \sigma .{\mathbf {\varvec{\kappa }}}[r.{ from }] - (f_1 + \dots + f_k) \end{aligned}$$

5.2 $$\begin{aligned}&(\tau ^k(\sigma )).\mathbf {g}= \sigma .\mathbf {g}+ (f_1 + \dots + f_k) \cdot r.\mathbf {u} \end{aligned}$$

5.3 $$\begin{aligned}&(\sigma .{\mathbf {\varvec{\kappa }}}, \sigma .\mathbf {g}+ f \cdot r.\mathbf {u}, \sigma .\mathbf {p}) \models r.\varphi ^{\mathrm {fall}}\wedge r.\varphi ^{\mathrm {rise}} \text{ for } \text{ all } f \in \{0, \dots , f_1 + \dots + f_k\} \end{aligned}$$

*Base case* $k = 1$. As schedule $\tau$ is applicable to $\sigma$, its first transition is enabled in $\sigma$. Thus, by the definition of an enabled transition, the rule *r* is unlocked, i.e., for all $f \in \{0, \dots , f_1\}$, it holds $(\sigma .{\mathbf {\varvec{\kappa }}}, \sigma .\mathbf {g}+ f_1 \cdot r.\mathbf {u}, \sigma .\mathbf {p}) \models r.\varphi ^{\mathrm {fall}}\wedge r.\varphi ^{\mathrm {rise}}$. By the definition, once the transition $\tau [1]$ is applied, it holds that $\tau ^1(\sigma ).{\mathbf {\varvec{\kappa }}}[{ from }] = \sigma .{\mathbf {\varvec{\kappa }}}[{ from }] - f_1$ and $(\tau ^k(\sigma )).\mathbf {g}= \sigma .\mathbf {g}+ f_1 \cdot r.\mathbf {u}$. Thus, Constraints (5.1)–(5.3) are satisfied for $k=1$.

*Inductive step* $k > 1$. As schedule $\tau$ is applicable to $\sigma$, its prefix $\tau ^k$ is applicable to $\sigma$. Hence, transition $\tau [k]$ is applicable to $\tau ^{k-1}(\sigma )$.

By the definition of an enabled transition, for all $f \in \{0, \dots , f_k\}$, it holds

$$\begin{aligned} ((\tau ^{k-1}(\sigma )).{\mathbf {\varvec{\kappa }}}, ((\tau ^{k-1}(\sigma )).\mathbf {g}+ f \cdot r.\mathbf {u}, \sigma .\mathbf {p}) \models r.\varphi ^{\mathrm {fall}}\wedge r.\varphi ^{\mathrm {rise}}. \end{aligned}$$

By applying the Eq. (5.2) for $k-1$ of the inductive hypothesis, we obtain that for all $f \in \{ 0, \dots , f_k \}$, it holds that $(\sigma .{\mathbf {\varvec{\kappa }}}, \sigma .\mathbf {g}+ (f_1 + \dots + f_{k-1} + f \cdot r.\mathbf {u}, \sigma .\mathbf {p}) \models r.\varphi ^{\mathrm {fall}}\wedge r.\varphi ^{\mathrm {rise}}$. By combining this constraint with the constraint (5.3) for $k-1$, we arrive at the constraint (5.3) for *k*.

By applying $\tau [k]$, we get that $(\tau ^k(\sigma )).{\mathbf {\varvec{\kappa }}}[r.{ from }] = (\tau ^{k-1}(\sigma )).{\mathbf {\varvec{\kappa }}}[r.{ from }] - f_k$ and $(\tau ^k(\sigma )).\mathbf {g}= (\tau ^{k-1}(\sigma )).\mathbf {g}+ f_k \cdot r.\mathbf {u}$. By applying (5.1) and (5.2) for $k-1$ to these equations, we arrive at the Eqs. (5.1) and (5.2) for *k*.

Based on (5.1) and (5.3) for all values of *k*, and in particular $k=m$, we can now show applicability. From Eq. (5.1), we immediately obtain that $\sigma .{\mathbf {\varvec{\kappa }}}[r.{ from }] \ge f_1 + \dots + f_m$. From constraint (5.3), we obtain that $(\sigma .{\mathbf {\varvec{\kappa }}}, \sigma .\mathbf {g}+ f \cdot r.\mathbf {u}, \sigma .\mathbf {p}) \models r.\varphi ^{\mathrm {fall}}\wedge r.\varphi ^{\mathrm {rise}}$ for all $f \in \{0, \dots , f_1 + \dots + f_m\}$. These are the required conditions for the transition $(r, f_1 + \dots + f_m)$ to be applicable to the configuration $\sigma$. $\square$

Consequently, when *c* has a single rule *r*, for configuration $\sigma$ and a schedule $\tau = (r, f_1), \ldots , (r, f_m)$, Lemma 5.1 allows us to take the singleton schedule (*r*, *f*) as $\mathsf {crep}^{\varOmega }_{c}[\sigma , \tau ]$ and to take the singleton schema $\{\varOmega \}\,r\,\{\varOmega \}$ as $\mathsf {cschema}^{\varOmega }_{c}$. The factor *f* is either $f_1 +\ldots + f_m$ or zero.

### Non-singleton looplet

Next we focus on non-singleton looplets. Thus, we assume that $|c| > 1$. Our construction is based on two directed trees, whose undirected versions are spanning trees, sharing the same root. In order to find a representative of a steady schedule $\tau$ which leads from $\sigma$ to $\tau (\sigma )$, we determine for each local state how many processes have to move in or out of the state, and then we move them along the edges of the trees. First, we give the definitions of such trees, and then we show how to use them to construct the representative schedules and the schema.

*Spanning out-trees and in-trees*

We construct the *underlying graph of looplet c*, that is, a directed graph $G_c$, whose vertices consist of local states that appear as components ${ from }$ or ${ to }$ of the rules from *c*, and the edges are the rules of *c*. More precisely, we construct a directed graph $G_c=(V_c, E_c, L_c)$, whose edges from $E_c$ are labeled by function $L_c: E_c \rightarrow c$ with the rules of *c* as follows:

$$\begin{aligned} V_c&= \{ \ell \mid \exists r \in c,\, r.{ to }= \ell \vee r.{ from }= \ell \},\\ E_c&= \{ (\ell , \ell ') \mid \exists r \in c,\, r.{ from }= \ell ,\, r.{ to }= \ell ' \}, \\ L_c((\ell , \ell '))&= r \text{, } \text{ if } r.{ from }= \ell ,\, r.{ to }= \ell ' \ \text{ for } (\ell , \ell ') \in E_c \text{ and } r \in c. \end{aligned}$$

#### Lemma 5.2

Given a threshold automaton and a non-singleton looplet $c \in {\mathcal R}/{\sim }$, graph $G_c$ is non-empty and strongly connected.

#### Proof

As, $|c| > 1$ and thus $E_c \ge 2$, graph $G_c$ is non-empty. To prove that $G_c$ is strongly connected, we consider a pair of rules $r_1, r_2 \in c$. By the definition of a looplet, it holds that $r_1 \prec ^+_{\scriptscriptstyle { P }}r_2$ and $r_2 \prec ^+_{\scriptscriptstyle { P }}r_1$. Thus, there is a path in $G_c$ from $r_1.{ to }$ to $r_2.{ from }$, and there is a path in $G_c$ from $r_2.{ to }$ to $r_1.{ from }$. As $r_1$ and $r_2$ correspond to some edges in $G_c$, there is a cycle that contains the vertices $r_1.{ from }$, $r_1.{ to }$, $r_2.{ from }$, and $r_2.{ to }$. Thus, graph $G_c$ is strongly connected. $\square$

As $G_c$ is non-empty and strongly connected, we can fix an arbitrary node $h \in V_c$ —called a *hub* —and construct two directed trees, whose undirected versions are spanning trees of the undirected version of $G_c$. These are two subgraphs of $G_c$: a directed tree $T_\mathsf {out}=(V_c, E_\mathsf {out})$, whose edges $E_\mathsf {out}\subseteq E_c$ are *pointing away from* *h* (out-tree); a directed tree $T_\mathsf {in}=(V_c, E_\mathsf {in})$, whose edges $E_\mathsf {in}\subseteq E_c$ are *pointing to* *h* (in-tree). For every node $v \in V_c {\setminus } \{h\}$, it holds that $|\{u :(u, v) \in E_\mathsf {out}\}|=1$ and $|\{w :(v, w) \in E_\mathsf {in}\}|=1$.

**Fig. 6:**
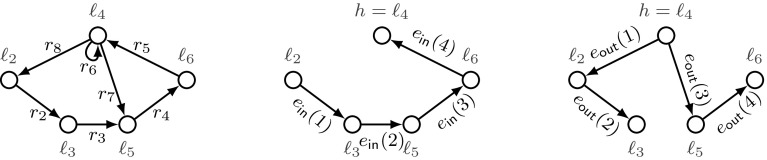
The underlying graph of the looplet $c_2$ of the threshold automaton from Example 3.3 and Fig. 3 (left), together with trees $T_\mathsf {in}$ (middle) and $T_\mathsf {out}$ (right)

Further, we fix a topological order $\preceq _\mathsf {in}$ on the edges of tree $T_\mathsf {in}$. More precisely, $\preceq _\mathsf {in}$ is such a partial order on $E_\mathsf {in}$ that for each pair of adjacent edges $(\ell , \ell '), (\ell ', \ell '') \in E_\mathsf {in}$, it holds that $(\ell , \ell ') \preceq _\mathsf {in}(\ell ', \ell '')$. In the same way, we fix a topological order $\preceq _\mathsf {out}$ on the edges of tree $T_\mathsf {out}$.

#### Example 5.1

Consider again the threshold automaton from Example 3.3 and Fig. 3. We construct trees $T_\mathsf {in}$ and $T_\mathsf {out}$ for looplet $c_2$, shown in Fig. 6.

Note that $V_c=\{\ell _2, \ell _3, \ell _4, \ell _5, \ell _6\}$, and $E_c=\{(\ell _2,\ell _3), (\ell _3,\ell _5), (\ell _5,\ell _6), (\ell _6,\ell _4),$ $(\ell _4, \ell _4), (\ell _4,\ell _5), (\ell _4,\ell _2)\}.$ Fix $\ell _4$ as a hub. We can fix a linear order $\preceq _\mathsf {in}$ such that $(\ell _2,\ell _3) \preceq _\mathsf {in}(\ell _3, \ell _5)\preceq _\mathsf {in}(\ell _5,\ell _6) \preceq _\mathsf {in}(\ell _6,\ell _4)$, and a linear order $\preceq _\mathsf {out}$ such that $(\ell _4,\ell _2) \preceq _\mathsf {out}(\ell _2, \ell _3)\preceq _\mathsf {out}(\ell _4,\ell _5)\preceq _\mathsf {out}(\ell _5,\ell _6)$.

Note that for the chosen hub $$ l_4$$ and this specific example, $$T_\mathsf {in}$$ and $$\preceq _\mathsf {in}$$ are uniquely defined, while an out-tree can be different from $$T_\mathsf {out}$$ from our Fig. 6 (the rules $$r_8, r_2, r_3, r_4$$ constitute a different tree from the same hub). Because out-tree $$T_\mathsf {out}$$ is not a chain, several linear orders different from $$\preceq _\mathsf {out}$$ can be chosen, e.g., $$(\ell _4,\ell _2)\preceq _\mathsf {out}(\ell _4,\ell _5) \preceq _\mathsf {out}(\ell _2, \ell _3)\preceq _\mathsf {out}(\ell _5,\ell _6)$$.

*Representatives of non-singleton looplets*

Using these trees, we show how to construct a representative $\mathsf {crep}^{\varOmega }_{c}[\sigma , \tau ]$ of a schedule $\tau$ applicable to $\sigma$ with $\sigma '=\tau (\sigma )$. For a configuration $\sigma$ and a schedule $\tau$ applicable to $\sigma$, consider the trees $T_\mathsf {in}$ and $T_\mathsf {out}$. We construct two sequences: the sequence $e_{\mathsf {in}}(1), \ldots , e_{\mathsf {in}}(|E_\mathsf {in}|)$ of all edges of $T_\mathsf {in}$ following the order $\preceq _\mathsf {in}$, i.e., if $e_{\mathsf {in}}(i)\preceq _\mathsf {in}e_{\mathsf {in}}(j)$, then $i\le j$; the sequence $e_{\mathsf {out}}(1), \ldots , e_{\mathsf {out}}(|E_\mathsf {out}|)$ of all edges of $T_\mathsf {out}$ following the order $\preceq _\mathsf {out}$. Further, we define the sequence of rules $r_\mathsf {in}(1), \ldots , r_\mathsf {in}(|E_\mathsf {in}|)$ with $r_\mathsf {in}(i) = L_c(e_\mathsf {in}(i))$ for $1 \le i \le |E_\mathsf {in}|$, and the sequence of rules $r_\mathsf {out}(1), \ldots , r_\mathsf {out}(|E_\mathsf {out}|)$ with $r_\mathsf {out}(i) = L_c(e_\mathsf {out}(i))$ for $1 \le i \le |E_\mathsf {out}|$. Using configurations $\sigma$ and $\sigma '=\tau (\sigma )$, we define:

$$\begin{aligned} \delta _{\mathsf {in}}(i)&= \sigma .{\mathbf {\varvec{\kappa }}}[f] - \sigma '.{\mathbf {\varvec{\kappa }}}[f] \text{, } \text{ for } f = r_\mathsf {in}(i).{ from } \text{ and } 1 \le i \le |E_\mathsf {in}|,\\ \delta _{\mathsf {out}}(j)&= \sigma '.{\mathbf {\varvec{\kappa }}}[t] - \sigma .{\mathbf {\varvec{\kappa }}}[t] \text{, } \text{ for } t = r_\mathsf {out}(j).{ to } \text{ and } 1 \le j \le |E_\mathsf {out}|. \end{aligned}$$

If $\delta _{\mathsf {in}}(i) \ge 0$, then $\delta _{\mathsf {in}}(i)$ processes should leave the local state $r_\mathsf {in}(i).{ from }$ towards the hub, and they do it exclusively using the edge $e_\mathsf {in}(i)$. If $\delta _{\mathsf {out}}(j) \ge 0$, then $\delta _{\mathsf {out}}(j)$ processes should reach the state $r_\mathsf {out}(j).{ to }$ from the hub, and they do it exclusively using the edge $e_\mathsf {out}(j)$. The negative values of $\delta _{\mathsf {in}}(i)$ and $\delta _{\mathsf {out}}(j)$ do not play any role in our construction, and thus, we use $\max (\delta _{\mathsf {in}}(i), 0)$ and $\max (\delta _{\mathsf {out}}(j), 0)$.

The main idea of the representative construction is as follows. First, we fire the sequence of rules $r_\mathsf {in}(1), \ldots , r_\mathsf {in}(k)$ to collect sufficiently many processes in the hub. Then, we fire the sequence of rules $r_\mathsf {out}(1), \ldots , r_\mathsf {out}(k)$ to distribute the required number of processes from the hub. As a result, for each location $\ell$ in the graph, the processes are transferred from $\ell$ to the other locations, if $\sigma [\ell ] > \sigma '[\ell ]$, and additional processes arrive at $\ell$, if $\sigma [\ell ] < \sigma '[\ell ]$. Using $\delta _\mathsf {in}(i)$ and $\delta _\mathsf {out}(i)$, we define the acceleration factors for each rule as follows:

$$\begin{aligned} w_{\mathsf {in}}(i)= & {} \sum \limits _{j:e_{\mathsf {in}}(j)\, \preceq _\mathsf {in}\, e_\mathsf {in}(i)} \max (\delta _{\mathsf {in}}(j), 0) \text{ and } \\ w_{\mathsf {out}}(i)= & {} \sum \limits _{j:e_{\mathsf {out}}(i)\, \preceq _\mathsf {out}\, e_\mathsf {out}(j)} \max (\delta _{\mathsf {out}}(j), 0). \end{aligned}$$

Finally, we construct the schedule $\mathsf {crep}^{\varOmega }_{c}[\sigma , \tau ]$ as follows:

5.4 $$\begin{aligned} \mathsf {crep}^{\varOmega }_{c}[\sigma , \tau ]= & {} (r_\mathsf {in}(1), w_{\mathsf {in}}(1)), \dots , (r_\mathsf {in}(|E_\mathsf {in}|), w_{\mathsf {in}}(|E_\mathsf {in}|)), \nonumber \\&(r_\mathsf {out}(1), w_{\mathsf {out}}(1)), \dots , (r_\mathsf {out}(|E_\mathsf {out}|), w_{\mathsf {out}}(|E_\mathsf {out}|)). \end{aligned}$$

**Fig. 7:**
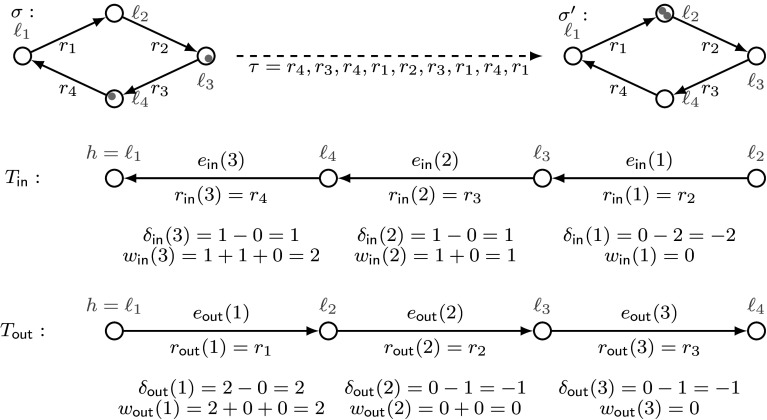
Construction of the representative of a schedule using the rules in the four-element looplet, following Example 5.2

#### Example 5.2

Consider the TA shown in Fig. 7. Let *c* be the four-element looplet that contains the rules $r_1$, $r_2$, $r_3$, and $r_4$, and $\tau$ be the schedule $\tau = (r_4,1),(r_3,1)$, $(r_4,1),(r_1,1),(r_2,1),(r_3,1),(r_1,1),(r_4,1),(r_1,1)$ that uses the rules of the looplet *c*. Consider a configuration $\sigma$ with $\sigma .{\mathbf {\varvec{\kappa }}}[\ell _3] =\sigma .{\mathbf {\varvec{\kappa }}}[\ell _4]= 1$, and $\sigma .{\mathbf {\varvec{\kappa }}}[\ell _1] =\sigma .{\mathbf {\varvec{\kappa }}}[\ell _2]= 0$. The final configuration $\sigma '=\tau (\sigma )$ has the following properties: $\sigma '.{\mathbf {\varvec{\kappa }}}[\ell _2]= 2$ and $\sigma '.{\mathbf {\varvec{\kappa }}}[\ell _1] = \sigma '.{\mathbf {\varvec{\kappa }}}[\ell _3] =\sigma '.{\mathbf {\varvec{\kappa }}}[\ell _4]= 0$. By comparing $\sigma$ and $\sigma '$, we notice that one process should move from $\ell _3$ to $\ell _2$, and one from $\ell _4$ to $\ell _2$. We will now show how this is achieved by our construction.

For constructing the representative schedule $\mathsf {crep}^{\varOmega }_{c}[\sigma , \tau ]$, we first define trees $T_\mathsf {in}$ and $T_\mathsf {out}$. If we chose $\ell _1$ to be the hub, we get that $E_\mathsf {in}= \{(\ell _4, \ell _1), (\ell _3,\ell _4), (\ell _2,\ell _3)\}$, and thus the order is $(\ell _2,\ell _3)\preceq _\mathsf {in}(\ell _3,\ell _4) \preceq _\mathsf {in}(\ell _4, \ell _1)$. Therefore, we obtain $e_{\mathsf {in}}(1) = (\ell _2,\ell _3)$, $e_{\mathsf {in}}(2) = (\ell _3,\ell _4)$ and $e_{\mathsf {in}}(3) = (\ell _4,\ell _1)$. By calculating $\delta _{\mathsf {in}}(i)$ for every $i\in \{1,2,3\}$, we see that $\delta _{\mathsf {in}}(2)=1$ and $\delta _{\mathsf {in}}(3)=1$ are positive. Consequently, two processes go to the hub: one from $r_\mathsf {in}(2).{ from }= \ell _3$ and one from $r_\mathsf {in}(3).{ from }=\ell _4$. The coefficients $w_\mathsf {in}$ give us acceleration factors for all rules.

Similarly, we obtain $E_\mathsf {out}= \{(\ell _1,\ell _2), (\ell _2,\ell _3), (\ell _3,\ell _4)\}$, and the order must be $(\ell _1,\ell _2) \preceq _\mathsf {out}(\ell _2,\ell _3)\preceq _\mathsf {out}(\ell _3,\ell _4)$. Thus, $e_{\mathsf {out}}(1) = (\ell _1,\ell _2)$, $e_{\mathsf {in}}(2) = (\ell _2,\ell _3)$, and $e_{\mathsf {out}}(3) = (\ell _3,\ell _4)$. Here only $\delta _{\mathsf {out}}(1)=2$ has a positive value, and hence, two processes should move from hub to the local state $r_\mathsf {out}(1).{ to }= \ell _2$. To achieve this, the acceleration factor of every rule $r_\mathsf {out}(i)$, $1\le i\le 3$, must be $w_\mathsf {out}(i)$.

Therefore, by Eq. (5.4), the representative schedule is

$$\begin{aligned} \mathsf {crep}^{\varOmega }_{c}[\sigma , \tau ] = (r_2,0), (r_3,1), (r_4,2), (r_1,2), (r_2,0), (r_3,0). \end{aligned}$$

Choosing another hub gives us another representative. For each hub, the representative is not longer than $2|c| = 8$, and leads to $\sigma '$ when applied to $\sigma$.

In the following, we fix a threshold automaton $\textsf {TA}$, a context $\varOmega$, and a non-singleton looplet *c* of the slice ${\textsf {TA}}|_{\varOmega }$. We also fix a configuration $\sigma$ of $\textsf {TA}$ and a schedule $\tau$ that is contained in *c* and is applicable to $\sigma$. Our goal is to prove Lemma 5.8, which states that $\mathsf {crep}^{\varOmega }_{c}[\sigma , \tau ]$ is indeed applicable to $\sigma$ and ends in $\tau (\sigma )$. To this end, we first prove auxiliary Lemmas 5.3–5.7.

#### Lemma 5.3

For every $i: 1 \le i \le |E_\mathsf {in}|$, it holds that $\sigma .{\mathbf {\varvec{\kappa }}}[r_i.{ from }] \ge \max (\delta _{\mathsf {in}}(i), 0)$, where $r_i = L_c(e_{\mathsf {in}}(i))$.

#### Proof

Recall that by the definition of a configuration, every counter $\sigma .{\mathbf {\varvec{\kappa }}}[\ell ]$ is non-negative. If $\delta _{\mathsf {in}}(i) \ge 0$, then $\max (\delta _{\mathsf {in}}(i), 0) = \delta _{\mathsf {in}}(i) = \sigma .{\mathbf {\varvec{\kappa }}}[r_i.{ from }] - \sigma '.{\mathbf {\varvec{\kappa }}}[r_i.{ from }]$, which is bound from above by $\sigma .{\mathbf {\varvec{\kappa }}}[r_i.{ from }]$. Otherwise, $\delta _{\mathsf {in}}(i) \le 0$, and we trivially have $\max (\delta _{\mathsf {in}}(i), 0) = 0$ and $0 \le \sigma .{\mathbf {\varvec{\kappa }}}[r_i.{ from }]$. $\square$

#### Lemma 5.4

Schedule $\tau _\mathsf {in}= (r_\mathsf {in}(1), w_{\mathsf {in}}(1)), \dots , (r_\mathsf {in}(|E_\mathsf {in}|), w_{\mathsf {in}}(|E_\mathsf {in}|))$ is applicable to configuration $\sigma$.

#### Proof

We denote by $\alpha ^i$ the schedule $(r_\mathsf {in}(1), w_{\mathsf {in}}(1)), \dots , (r_\mathsf {in}(i), w_{\mathsf {in}}(i))$, for $1 \le i \le |E_\mathsf {in}|$. Then $\tau _\mathsf {in}=\alpha ^{|E_\mathsf {in}|}$.

All rules $r_\mathsf {in}(1), \ldots , r_\mathsf {in}(|E_\mathsf {in}|)$ are from ${{\mathcal R}}|_{\varOmega }$, and thus are unlocked. Hence, it is sufficient to show that the values of the locations from the set $V_c$ are large enough to enable each transition $(r_{\mathsf {in}}(i), w_{\mathsf {in}}(i))$ for $1 \le i \le |E_\mathsf {in}|$. To this end, we prove by induction that $(\alpha ^{i-1}(\sigma )).{\mathbf {\varvec{\kappa }}}[r_i.{ from }] \ge w_{\mathsf {in}}(i)$, for $1 \le i \le |E_\mathsf {in}|$ and $r_i= L_c(e_{\mathsf {in}}(i))$.

*Base case* $i=1$. For $r_1 = L_c(e_{\mathsf {in}}(1))$, we want to show that $\sigma .{\mathbf {\varvec{\kappa }}}[r_1.{ from }] \ge w_{\mathsf {in}}(1)$. As $e_\mathsf {in}(1)$ is the first element of the sequence $e_\mathsf {in}(1), \dots , e_\mathsf {in}(E_\mathsf {in})$, which respects the order $\preceq _\mathsf {in}$, we conclude that $w_{\mathsf {in}}(1) = \max (\delta _{\mathsf {in}}(1), 0)$. From Lemma 5.3, it follows that $\sigma .{\mathbf {\varvec{\kappa }}}[r_1.{ from }] \ge \max (\delta _{\mathsf {in}}(1), 0)$.

*Inductive step k* assume that for all $i: 1 \le i \le k - 1 < |E_\mathsf {in}|$, schedule $\alpha ^i$ is applicable to $\sigma$ and show that $(\alpha ^{k-1}(\sigma )).{\mathbf {\varvec{\kappa }}}[r_k.{ from }] \ge w_{\mathsf {in}}(k)$ with $r_k = L_c(e_{\mathsf {in}}(k))$.

To this end, we construct the set of edges $P_k$ that precede the edge $e_{\mathsf {in}}(k)$ in the topological order $\preceq _\mathsf {in}$, that is, $P_k = \{ e \mid e \in E_\mathsf {in},\ e \preceq _\mathsf {in}e_{\mathsf {in}}(k),\ e \ne e_{\mathsf {in}}(k) \}$. We show that the following equation holds:

5.5 $$\begin{aligned} \alpha ^{k-1}(\sigma )).{\mathbf {\varvec{\kappa }}}[r_k.{ from }] = \sigma .{\mathbf {\varvec{\kappa }}}[r_k.{ from }] + \sum \limits _{e_{\mathsf {in}}(j) \in P_k} \max (\delta _{\mathsf {in}}(j), 0). \end{aligned}$$

Indeed, if one picks an edge $e_{\mathsf {in}}(j) \in P_k$, the edge $e_{\mathsf {in}}(j)$ adds $w_{\mathsf {in}}(j)$ to the counter ${\mathbf {\varvec{\kappa }}}[r_k.{ from }]$. As the sequence $\{e_{\mathsf {in}}(i)\}_{i\le k}$ is topologically sorted, it follows that $j <k$. Moreover, as the tree $T_{\mathsf {in}}$ is oriented towards the root, $e_{\mathsf {in}}(k)$ is the only edge leaving the local state $r_k.{ from }$. Thus, no edge $e_{\mathsf {in}}(i)$ with $i < k$ decrements the counter $\sigma .{\mathbf {\varvec{\kappa }}}[r_k.{ from }]$.

From Eq. (5.5) and Lemma 5.3, we conclude that $(\alpha ^{k-1}(\sigma )).{\mathbf {\varvec{\kappa }}}[r_k.{ from }]$ is not less than $\max (\delta _{\mathsf {in}}(k), 0) + \sum _{e_{\mathsf {in}}(j) :e_{\mathsf {in}}(j)\, \preceq _\mathsf {in}\, e_{\mathsf {in}}(k),\ j \ne k} \max (\delta _{\mathsf {in}}(j), 0)$, which equals to $w_{\mathsf {in}}(k)$. This proves the inductive step.

Therefore, we have shown that $\tau _\mathsf {in}= \alpha ^{|E_\mathsf {in}|}$ is applicable to $\sigma$. $\square$

The following lemma is easy to prove by induction on the length of a schedule. The base case for a single transition follows from the definition of a counter system.

#### Lemma 5.5

Let $\sigma$ and $\sigma '$ be two configurations and $\tau$ be a schedule applicable to $\sigma$ such that $\tau (\sigma ) = \sigma '$. Then it holds that $\sum _{\ell \in {\mathcal L}} (\sigma '[\ell ] - \sigma [\ell ]) = 0$.

Further, we show that the required number of processes is reaching (or leaving) the hub, when the transitions derived from the trees $T_\mathsf {in}$ and $T_\mathsf {out}$ are executed:

#### Lemma 5.6

The following equality holds:

$$\begin{aligned} \sigma '.{\mathbf {\varvec{\kappa }}}[h] - \sigma .{\mathbf {\varvec{\kappa }}}[h] = \sum _{1 \le i \le |E_\mathsf {in}|} \max (\delta _{\mathsf {in}}(i), 0) - \sum _{1 \le i \le |E_\mathsf {out}|} \max (\delta _{\mathsf {out}}(i), 0). \end{aligned}$$

#### Proof

Recall that $T_\mathsf {in}$ is a tree directed towards *h*, and the undirected version of $T_\mathsf {in}$ is a spanning tree of graph *C*. Hence, for each local state $\ell \in V_c {\setminus } \{h\}$, there is exactly one edge $e \in E_\mathsf {in}$ with $L_c(e).{ from }= \ell$. Thus, the following equation holds:

5.6 $$\begin{aligned} \sum _{1 \le i \le |E_\mathsf {in}|} \max (\delta _{\mathsf {in}}(i), 0) = \sum _{\ell \in V_c {\setminus } \{h\}} \max (\sigma .{\mathbf {\varvec{\kappa }}}[\ell ] - \sigma '.{\mathbf {\varvec{\kappa }}}[\ell ], 0). \end{aligned}$$

Similarly, $T_\mathsf {out}$ is a tree directed outwards *h*, and the undirected version of $T_\mathsf {out}$ is a spanning tree of graph *C*. Hence, for each local state $\ell \in V_c {\setminus } \{h\}$, there is exactly one edge $e \in E_\mathsf {out}$ with $L_c(e).{ to }= \ell$. Thus, the following equation holds:

5.7 $$\begin{aligned} \sum _{1 \le i \le |E_\mathsf {out}|} \max (\delta _{\mathsf {out}}(i), 0) = \sum _{\ell \in V_c {\setminus } \{h\}} \max (\sigma '.{\mathbf {\varvec{\kappa }}}[\ell ] - \sigma .{\mathbf {\varvec{\kappa }}}[\ell ], 0). \end{aligned}$$

By combining (5.6) and (5.7), we obtain the following:

5.8 $$\begin{aligned}&\sum _{1 \le i \le |E_\mathsf {in}|} \max (\delta _{\mathsf {in}}(i), 0) - \sum _{1 \le i \le |E_\mathsf {out}|} \max (\delta _{\mathsf {out}}(i), 0) \nonumber \\&\quad =\sum _{\ell \in V_c {\setminus } \{h\}} \big ( \max (\sigma .{\mathbf {\varvec{\kappa }}}[\ell ] - \sigma '.{\mathbf {\varvec{\kappa }}}[\ell ], 0) - \max (\sigma '.{\mathbf {\varvec{\kappa }}}[\ell ] - \sigma .{\mathbf {\varvec{\kappa }}}[\ell ], 0) \big )\nonumber \\&\quad =\sum _{\ell \in V_c {\setminus } \{h\}} \big (\sigma .{\mathbf {\varvec{\kappa }}}[\ell ] - \sigma '.{\mathbf {\varvec{\kappa }}}[\ell ]\big ) =\left( \sum _{\ell \in V_c} \sigma .{\mathbf {\varvec{\kappa }}}[\ell ] - \sigma '.{\mathbf {\varvec{\kappa }}}[\ell ]\right) - \big (\sigma .{\mathbf {\varvec{\kappa }}}[h] - \sigma '.{\mathbf {\varvec{\kappa }}}[h]\big ). \end{aligned}$$

As the initial schedule $\tau$ is applicable to $\sigma$, and $\tau (\sigma ) = \sigma '$, by Lemma 5.5, $\sum _{\ell \in {\mathcal L}} (\sigma .{\mathbf {\varvec{\kappa }}}[\ell ] - \sigma '.{\mathbf {\varvec{\kappa }}}[\ell ]) = 0$. As all rules in $\mathsf {crep}^{\varOmega }_{c}[\sigma , \tau ]$ are from ${{\mathcal R}}|_{\varOmega }$ and thus change only the counters of local states in $V_c$, for each local state $\ell \in {\mathcal L}{\setminus } V_c$, its respective counter does not change, that is, $\sigma .{\mathbf {\varvec{\kappa }}}[\ell ] - \sigma '.{\mathbf {\varvec{\kappa }}}[\ell ] = 0$. Hence, $\sum _{\ell \in V_c} (\sigma .{\mathbf {\varvec{\kappa }}}[\ell ] - \sigma '.{\mathbf {\varvec{\kappa }}}[\ell ]) = 0$. From this and Eq. (5.8), the statement of the lemma follows. $\square$

#### Lemma 5.7

If $\tau _\mathsf {in}$ denotes the schedule $(r_\mathsf {in}(1), w_{\mathsf {in}}(1)), \dots , (r_\mathsf {in}(|E_\mathsf {in}|), w_{\mathsf {in}}(|E_\mathsf {in}|))$, the following equation holds:

$$\begin{aligned} \tau _\mathsf {in}(\sigma ).{\mathbf {\varvec{\kappa }}}[\ell ]= {\left\{ \begin{array}{ll} \sigma '.{\mathbf {\varvec{\kappa }}}[h] + \sum _{1 \le i \le |E_\mathsf {out}|} \max (\delta _{\mathsf {out}}(i),0), &{} \quad \text {if }\,\ell = h\\ \min (\sigma .{\mathbf {\varvec{\kappa }}}[\ell ],\sigma '.{\mathbf {\varvec{\kappa }}}[\ell ]), &{}\quad \text {if }\,\ell \in V_c{\setminus } \{h\}. \end{array}\right. } \end{aligned}$$

#### Proof

We prove the lemma by case distinction:

*Case* $\ell = h$ We show that $(\tau _\mathsf {in}(\sigma )).{\mathbf {\varvec{\kappa }}}[h] = \sigma .{\mathbf {\varvec{\kappa }}}[h] + \sum _{1 \le i \le |E_\mathsf {in}|} \max (\delta _{\mathsf {in}}(i), 0)$. Indeed, let *P* be the indices of edges coming into *h*, i.e., $P = \{ i \mid 1 \le i \le |E_\mathsf {in}|,\ L_c(e_{\mathsf {in}}(i)) = r,\ h = r.{ to }\}$. As all edges in $T_\mathsf {in}$ are oriented towards *h*, it holds that $(\tau _\mathsf {in}(\sigma )).{\mathbf {\varvec{\kappa }}}[h]$ equals to $\sigma .{\mathbf {\varvec{\kappa }}}[h] + \sum _{i \in P} w_{\mathsf {in}}(i)$. By unfolding the definition of $w_\mathsf {in}$, we obtain that $(\tau _\mathsf {in}(\sigma )).{\mathbf {\varvec{\kappa }}}[h] = \sigma .{\mathbf {\varvec{\kappa }}}[h] + \sum _{1 \le i \le |E_\mathsf {in}|} \max (\delta _{\mathsf {in}}(i), 0)$. We observe that by Lemma 5.6, this sum equals to $\sigma '.{\mathbf {\varvec{\kappa }}}[h] + \sum _{1 \le i \le |E_\mathsf {out}|} \max (\delta _{\mathsf {out}}(i), 0)$. This proves the first case.

*Case* $\ell \in V_c {\setminus } \{h\}$ We show that $(\tau _\mathsf {in}(\sigma )).{\mathbf {\varvec{\kappa }}}[\ell ] = \min (\sigma .{\mathbf {\varvec{\kappa }}}[\ell ], \sigma '.{\mathbf {\varvec{\kappa }}}[\ell ])$. Indeed, fix a node $\ell \in V_c {\setminus } \{h\}$ and construct two sets: the set of incoming edges $In = \{e_{\mathsf {in}}(i) \mid \exists \ell ' \in V_c.\ e_{\mathsf {in}}(i) = (\ell ', \ell ) \}$ and the singleton set of outgoing edges $Out = \{e_{\mathsf {in}}(i) \mid \exists \ell ' \in V_c.\ e_{\mathsf {in}}(i) = (\ell , \ell ') \}$. By summing up the effect of all transitions in $\tau _\mathsf {in}$, we obtain $(\tau _\mathsf {in}(\sigma )).{\mathbf {\varvec{\kappa }}}[\ell ] = \sigma .{\mathbf {\varvec{\kappa }}}[\ell ] + \sum _{e_{\mathsf {in}}(i) \in In } w_{\mathsf {in}}(i) - \sum _{e_{\mathsf {out}}(i) \in Out } w_{\mathsf {out}}(i)$. By unfolding the definition of $w_\mathsf {in}$, we obtain $(\tau _\mathsf {in}(\sigma )).{\mathbf {\varvec{\kappa }}}[\ell ] = \sigma .{\mathbf {\varvec{\kappa }}}[\ell ] - \sum _{e_{\mathsf {in}}(i) \in Out } \delta _{\mathsf {in}}(i)$, which can be rewritten as $\sigma .{\mathbf {\varvec{\kappa }}}[\ell ] - \max (\sigma .{\mathbf {\varvec{\kappa }}}[\ell ] - \sigma '.{\mathbf {\varvec{\kappa }}}[\ell ], 0)$, which, in turn, equals to $\min (\sigma .{\mathbf {\varvec{\kappa }}}[\ell ], \sigma '.{\mathbf {\varvec{\kappa }}}[\ell ])$. This proves the second case. $\square$

Now we are in a position to prove that schedule $\mathsf {crep}^{\varOmega }_{c}[\sigma , \tau ]$ is applicable to configuration $\sigma$ and results in configuration $\tau (\sigma )$:

#### Lemma 5.8

The schedule $\mathsf {crep}^{\varOmega }_{c}[\sigma , \tau ]$ has the following properties: (a) $\mathsf {crep}^{\varOmega }_{c}[\sigma , \tau ]$ is applicable to $\sigma$, and (b) $\mathsf {crep}^{\varOmega }_{c}[\sigma , \tau ]$ results in $\tau (\sigma )$ when applied to $\sigma$.

#### Proof

Denote with $\tau _\mathsf {in}$ the prefix $(r_\mathsf {in}(1), w_{\mathsf {in}}(1)), \ldots , (r_\mathsf {in}(|E_\mathsf {in}|), w_{\mathsf {in}}(|E_\mathsf {in}|))$ of the schedule $\mathsf {crep}^{\varOmega }_{c}[\sigma , \tau ]$. For each $j: 1 \le j \le |E_\mathsf {out}|$, denote with $\beta ^j$ the prefix of $\mathsf {crep}^{\varOmega }_{c}[\sigma , \tau ]$ that has length of $|E_\mathsf {in}| + j$. Note that $\beta ^{|E_\mathsf {out}|} = \mathsf {crep}^{\varOmega }_{c}[\sigma , \tau ]$.

*Proving applicability of* $\mathsf {crep}^{\varOmega }_{c}[\sigma , \tau ]$ to $\sigma$ We notice that all rules in $\mathsf {crep}^{\varOmega }_{c}[\sigma , \tau ]$ are from ${{\mathcal R}}|_{\varOmega }$ and thus are unlocked, and that $\tau _\mathsf {in}$ is applicable to $\sigma$ by Lemma 5.4. Hence, we only have to check that the values of counters from $V_c$ are large enough, so that transitions $(r_{\mathsf {out}}(j), w_{\mathsf {out}}(j))$ can fire.

We prove that each schedule $\beta ^j$ is applicable to $\sigma$, for $j: 1 \le j \le |E_\mathsf {out}|$. We do so by induction on the distance from the root *h* in the tree $T_\mathsf {out}$.

*Base case* root node *h*. Denote with $O_h$ the set $\{(\ell , \ell ') \in E_\mathsf {out}\mid \ell = h \}$. Let $j_1, \dots , j_m$ be the indices of all edges in $O_h$, and $j_m$ be the maximum among them.

From Lemma 5.7, $(\tau _\mathsf {in}(\sigma )).{\mathbf {\varvec{\kappa }}}[h] = \sigma '.{\mathbf {\varvec{\kappa }}}[h] + \sum _{1 \le i \le |E_\mathsf {out}|} \max (\delta _{\mathsf {out}}(i), 0) = \sigma '.{\mathbf {\varvec{\kappa }}}[h] + \sum _{e_{\mathsf {out}}(j) \in O_h} w_{\mathsf {out}}(j)$. Thus, every transition $(e_{\mathsf {out}}(j), w_{\mathsf {out}}(j))$ with $e_{\mathsf {out}}(j)\in O_h$, is applicable to $\beta ^{j-1}(\sigma )$. Also, $(\beta ^{j_m}(\sigma )).{\mathbf {\varvec{\kappa }}}[h] = \sigma '.{\mathbf {\varvec{\kappa }}}[h]$.

*Inductive step* assume that for a node $\ell \in V_c$ and an edge $e_{\mathsf {out}}(k) = (\ell , \ell ') \in E_\mathsf {out}$ outgoing from node $\ell$, schedule $\beta ^k$ is applicable to configuration $\sigma$. Show that for each edge $e_{\mathsf {out}}(i)$ outgoing from node $\ell '$ the following hold: (i) schedule $\beta ^i$ is also applicable to $\sigma$; and (ii) $\beta ^{|E_\mathsf {out}|}(\sigma ).{\mathbf {\varvec{\kappa }}}[\ell '] = \sigma '.{\mathbf {\varvec{\kappa }}}[\ell ']$.

(i) As the sequence $\{e_{\mathsf {out}}(j)\}_{j\le |E_\mathsf {out}|}$ is topologically sorted, for each edge $e_{\mathsf {out}}(i)$ outgoing from node $\ell '$, it holds that $k < i$.

From Lemma 5.7, we have that $\beta ^k(\sigma ).{\mathbf {\varvec{\kappa }}}[\ell '] = \min (\sigma .{\mathbf {\varvec{\kappa }}}[\ell '], \sigma '.{\mathbf {\varvec{\kappa }}}[\ell '])$. Because the transition $(e_{\mathsf {out}}(k), w_{\mathsf {out}}(k))$ adds $w_{\mathsf {out}}(k)$ to $\beta ^{k-1}(\sigma ).{\mathbf {\varvec{\kappa }}}[\ell ']$, we have $\beta ^k(\sigma ).{\mathbf {\varvec{\kappa }}}[\ell '] = \min (\sigma .{\mathbf {\varvec{\kappa }}}[\ell '], \sigma '.{\mathbf {\varvec{\kappa }}}[\ell ']) + w_{\mathsf {out}}(k)$. Let *S* be the set of all immediate successors of $e_\mathsf {out}(k)$, i.e., $S = \{ i \mid \exists \ell ''.\ (\ell ', \ell '') = e_\mathsf {out}(i) \}$. From the definition of $w_{\mathsf {out}}(k)$, it follows that $w_{\mathsf {out}}(k) = \max (\delta _{\mathsf {out}}(k), 0) + \sum _{s \in S} w_{\mathsf {out}}(s)$. Thus, the transition $(e_{\mathsf {out}}(i), w_{\mathsf {out}}(i))$ for edge $e_{\mathsf {out}}(i)$ outgoing from node $\ell '$, can be executed.

(ii) Let $j_1, \dots , j_m$ be the indices of all edges outgoing from $\ell '$, and $j_m$ be the maximum among them. From (i), it follows that

$$\begin{aligned} (\beta ^{j_m}(\sigma )).{\mathbf {\varvec{\kappa }}}[\ell '] = \min (\sigma .{\mathbf {\varvec{\kappa }}}[\ell '], \sigma '.{\mathbf {\varvec{\kappa }}}[\ell ']) + \max (\delta _{\mathsf {out}}(k), 0), \end{aligned}$$

which equals to $\sigma '.{\mathbf {\varvec{\kappa }}}[\ell ']$.

This proves that the schedule $\beta ^{|E_\mathsf {out}|}=\mathsf {crep}^{\varOmega }_{c}[\sigma , \tau ]$ is applicable to $\sigma$.

*Proving that* $\mathsf {crep}^{\varOmega }_{c}[\sigma , \tau ]$ *results in* $\tau (\sigma )$ From the induction above, we conclude that for each $\ell \in V_c$, it holds that $(\beta ^{|E_\mathsf {out}|}(\sigma )).{\mathbf {\varvec{\kappa }}}[\ell ] = \sigma '.{\mathbf {\varvec{\kappa }}}[\ell ]$. Edges in the trees $T_\mathsf {in}$ and $T_\mathsf {out}$ change only local states from $V_c$. We conclude that for all $\ell \in {\mathcal L}$, it holds that $\mathsf {crep}^{\varOmega }_{c}[\sigma , \tau ](\sigma ).{\mathbf {\varvec{\kappa }}}[\ell ] = \sigma '.{\mathbf {\varvec{\kappa }}}[\ell ]$. As the rules in non-singleton looplets do not change shared variables, $\mathsf {crep}^{\varOmega }_{c}[\sigma , \tau ](\sigma ).\mathbf {g}= \sigma .\mathbf {g}= \sigma '.\mathbf {g}$. Therefore, $\mathsf {crep}^{\varOmega }_{c}[\sigma , \tau ](\sigma ) = \sigma '$. $\square$

### Representatives for one context and one looplet

We now summarize results from Sects. 5.1 and 5.2, giving the representative of a schedule $\tau$ in the case when $\tau$ uses only the rules from one looplet, and does not change its context. If the given looplet consists of a single rule, the construction is given in Sect. 5.1, and otherwise in Sect. 5.2. We show that these constructions indeed give us a schedule of bounded length, that reaches the same state as $\tau$.

In the following, given a threshold automaton $\textsf {TA}$ and a looplet *c*, we will say that a *schedule* $\tau =t_1, \dots , t_n$ *is contained in* *c*, if $[{t_i.{ rule }}] = c$ for $1 \le i \le n$.

#### Theorem 5.1

Fix a threshold automaton, and a context $\varOmega$, and a looplet *c* in the slice ${\textsf {TA}}|_{\varOmega }$. Let $\sigma$ be a configuration and $\tau$ be a steady schedule contained in *c* and applicable to $\sigma$. There exists a representative schedule $\mathsf {crep}^{\varOmega }_{c}[\sigma , \tau ]$ with the following properties:

- schedule $\mathsf {crep}^{\varOmega }_{c}[\sigma , \tau ]$ is applicable to $\sigma$, and $\mathsf {crep}^{\varOmega }_{c}[\sigma , \tau ](\sigma ) = \tau (\sigma )$,
- the rule of each transition *t* in $\mathsf {crep}^{\varOmega }_{c}[\sigma , \tau ]$ belongs to *c*, that is, $[{t.{ rule }}] = c$,
- schedule $\mathsf {crep}^{\varOmega }_{c}[\sigma , \tau ]$ is not longer than $2 \cdot |c|$.

#### Proof

If $|c|=1$, then we use a single accelerated transition or the empty schedule as representative, as described in Lemma 5.1.

If $|c| > 1$, we construct the representative as in Sect. 5.2, so that by Lemma 5.8 property (a) follows. For every edge $e \in E_c$, the rule $L_c(e)$ belongs to *c*, and thus $\mathsf {crep}^{\varOmega }_{c}[\sigma , \tau ]$ satisfies property (b). As $|E_\mathsf {in}| \le |c|$ and $|E_\mathsf {out}| \le |c|$, we conclude that $|\mathsf {crep}^{\varOmega }_{c}[\sigma , \tau ]| \le 2 \cdot |c|$, and thus property c) is also satisfied. From this and Lemma 5.8, we conclude that $\mathsf {crep}^{\varOmega }_{c}[\sigma , \tau ]$ is the required representative schedule. $\square$

Theorem 5.1 gives us a way to construct schemas that generate all representatives of the schedules contained in a looplet:

#### Theorem 5.2

Fix a threshold automaton $\textsf {TA}$, a context $\varOmega$, and a looplet *c* in the slice ${\textsf {TA}}|_{\varOmega }$. There exists a schema $\mathsf {cschema}^{\varOmega }_{c}$ with the following properties:

Fix an arbitrary configuration $\sigma$ and a steady schedule $\tau$ that is contained in *c* and is applicable to $\sigma$. Let $\tau '=\mathsf {crep}^{\varOmega }_{c}[\sigma , \tau ]$ be the representative schedule of $\tau$, from Theorem 5.1. Then, $\textsf {path}(\sigma , \tau ')$ is generated by $\mathsf {cschema}^{\varOmega }_{c}$. Moreover, the length of $\mathsf {cschema}^{\varOmega }_{c}$ is at most $2 \cdot |c|$.

#### Proof

Note that $\tau '=\mathsf {crep}^{\varOmega }_{c}[\sigma , \tau ]$ can be constructed in two different ways depending on the looplet *c*.

If $|c|=1$, then by Lemma 5.1 we have that $\tau '=(r,f)$ for a rule $r\in c$ and a factor $f\in {\mathbb N}_0$. In this case we construct $\mathsf {cschema}^{\varOmega }_{c}$ to be

$$\begin{aligned} \mathsf {cschema}^{\varOmega }_{c}=\{\varOmega \}\,r\,\{\varOmega \}. \end{aligned}$$

It is easy to see that $\textsf {path}(\sigma , \tau ')$ is generated by $\mathsf {cschema}^{\varOmega }_{c}$, as well as that the length of $\mathsf {cschema}^{\varOmega }_{c}$ is exactly 1, that is less than $2 \cdot |c|$.

If $|c|>1$, then we use the trees $T_\mathsf {in}$ and $T_\mathsf {out}$ to construct the schema $\mathsf {cschema}^{\varOmega }_{c}$ as follows:

5.9 $$\begin{aligned} \mathsf {cschema}^{\varOmega }_{c} = \{\varOmega \}\,r_{\mathsf {in}}(1) \cdots r_{\mathsf {in}}(|E_\mathsf {in}|) \cdot r_{\mathsf {out}}(1) \cdots r_{\mathsf {out}}(|E_\mathsf {out}|)\,\{\varOmega \}. \end{aligned}$$

Since for an arbitrary configuration $\sigma$ and a schedule $\tau$, we use the same sequence of edges in Eqs. (5.4) and (5.9) to construct $\mathsf {crep}^{\varOmega }_{c}[\sigma , \tau ]$ and $\mathsf {cschema}^{\varOmega }_{c}$, the schema $\mathsf {cschema}^{\varOmega }_{c}$ generates all paths of the representative schedules, and its length is at most $2 \cdot |c|$. $\square$

## Case II: one context and multiple looplets

In this section, we show that for each steady schedule, there exists a representative steady schedule of bounded length that reaches the same final state.

### Theorem 6.1

Fix a threshold automaton and a context $\varOmega$. For every configuration $\sigma$ with $\omega (\sigma ) = \varOmega$ and every steady schedule $\tau$ applicable to $\sigma$, there exists a steady schedule $\mathsf {srep}_{\varOmega }[\sigma ,\tau ]$ with the following properties:

- $\mathsf {srep}_{\varOmega }[\sigma ,\tau ]$ is applicable to $\sigma$, and $\mathsf {srep}_{\varOmega }[\sigma ,\tau ](\sigma ) = \tau (\sigma )$,
- $$|\mathsf {srep}_{\varOmega }[\sigma ,\tau ]| \le 2 \cdot |({{\mathcal R}}|_{\varOmega })|$$

To construct a representative schedule, we fix a context $\varOmega$ of a TA, a configuration $\sigma$ with $\omega (\sigma ) = \varOmega$, and a steady schedule $\tau$ applicable to $\sigma$. The key notion in our construction is a projection of a schedule on a set of looplets:

### Definition 6.1

Let $\tau = t_1, \ldots , t_k$, for $k>0$, be a schedule, and let *C* be a set of looplets. Given an increasing sequence of indices $i(1), \ldots , i(m) \in \{1, \ldots , k \}$, where $m\le k$, i.e., $i(j) < i(j+1)$, for $1 \le j < m$, a schedule $t_{i(1)} \ldots t_{i(m)}$ is a projection of $\tau$ on *C*, if each index $j \in \{ 1, \ldots , k \}$ belongs to $\{ i(1), \ldots , i(m) \}$ if and only if $[{t_j.{ rule }}] \in C$.

In fact, each schedule $\tau$ has a unique projection on a set *C*. In the following, we write ${\tau }|_{c_1,\ldots ,c_m}$ to denote the projection of $\tau$ on a set $\{c_1,\ldots ,c_m\}$.

Provided that $c_1, \ldots , c_m$ are all looplets of the slice ${{\mathcal R}}|_{\varOmega }$ ordered with respect to , we construct the following sequences of projections on each looplet (note that $\pi _0$ is the empty schedule): $\pi _i = {\tau }|_{c_1} \cdot \cdots \cdot {\tau }|_{c_i} \text{ for } 0 \le i \le m$.

Having defined $\{\pi _i\}_{0 \le i \le m}$, we construct the representative $\mathsf {srep}_{\varOmega }[\sigma ,\tau ]$ simply as a concatenation of the representatives of each looplet:

$$\begin{aligned} \mathsf {srep}_{\varOmega }[\sigma ,\tau ] = \mathsf {crep}^{\varOmega }_{c_1}[\pi _0(\sigma ), {\tau }|_{c_1}] \cdot \mathsf {crep}^{\varOmega }_{c_2}[\pi _1(\sigma ), {\tau }|_{c_2}] \cdot \ldots \cdot \mathsf {crep}^{\varOmega }_{c_m}[\pi _{m-1}(\sigma ), {\tau }|_{c_m}] \end{aligned}$$

**Fig. 8:**
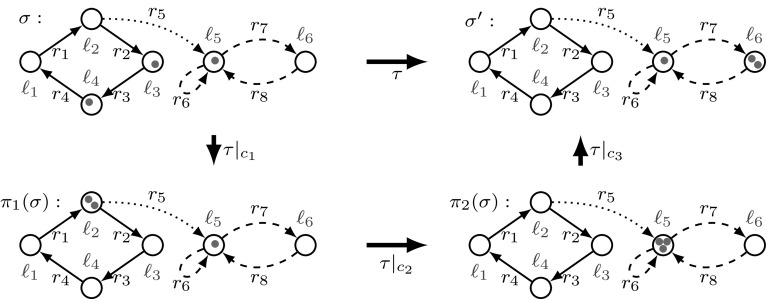
Threshold automaton and configurations used in Example 6.1

### Example 6.1

Consider the TA shown in Fig. 8. It has three looplets, namely $c_1 = \{r_1, r_2,r_3,r_4\}$, $c_2 = \{r_5\}$, $c_3=\{r_6, r_7, r_8\}$, and the rules are depicted as solid, dotted, and dashed, respectively. These looplets are ordered such that .

Let $\sigma$ be the configuration represented in Fig. 8 left, i.e. ${\mathbf {\varvec{\kappa }}}[\ell _3]= {\mathbf {\varvec{\kappa }}}[\ell _4] = {\mathbf {\varvec{\kappa }}}[\ell _5]=1$ and ${\mathbf {\varvec{\kappa }}}[\ell _3]={\mathbf {\varvec{\kappa }}}[\ell _4] = {\mathbf {\varvec{\kappa }}}[\ell _5]=0$. Let $\tau$ be the schedule $(r_4,1),(r_6,1),(r_3,1),$ $(r_4,1),(r_1,1),(r_2,1),(r_7,1),(r_3,1),(r_1,1),(r_5,1), (r_7,1),(r_4,1),(r_8,1),(r_1,1),(r_6,1),$ $(r_7,1),(r_5,1),(r_8,1),(r_7,1)$. Note that $\tau$ is applicable to $\sigma$ and that $\tau (\sigma )$ is the configuration $\sigma '$ from Fig. 8 right, i.e. ${\mathbf {\varvec{\kappa }}}[\ell _5]=1$, ${\mathbf {\varvec{\kappa }}}[\ell _6]=2$ and ${\mathbf {\varvec{\kappa }}}[\ell _1]={\mathbf {\varvec{\kappa }}}[\ell _2] ={\mathbf {\varvec{\kappa }}}[\ell _3]= {\mathbf {\varvec{\kappa }}}[\ell _4] =0$. We construct the representative schedule $\mathsf {srep}_{\varOmega }[\sigma ,\tau ]$.

Projection of $\tau$ on the looplets $c_1$, $c_2$, and $c_3$, gives us the following schedules:

$$\begin{aligned} {\tau }|_{c_1}&= (r_4,1),(r_3,1),(r_4,1),(r_1,1),(r_2,1), (r_3,1),(r_1,1),(r_4,1),(r_1,1), \\ {\tau }|_{c_2}&= (r_5,1), (r_5,1), \\ {\tau }|_{c_3}&= (r_6,1),(r_7,1),(r_7,1),(r_8,1),(r_6,1),(r_7,1),(r_8,1),(r_7,1). \end{aligned}$$

Recall that

$$\begin{aligned} \mathsf {srep}_{\varOmega }[\sigma ,\tau ] = \mathsf {crep}^{\varOmega }_{c_1}[\pi _0(\sigma ), {\tau }|_{c_1}] \cdot \mathsf {crep}^{\varOmega }_{c_2}[\pi _1(\sigma ), {\tau }|_{c_2}] \cdot \mathsf {crep}^{\varOmega }_{c_3}[\pi _{2}(\sigma ), {\tau }|_{c_3}]. \end{aligned}$$

In order to construct this schedule, we firstly construct the required configurations. Note that $\pi _0(\sigma )=\sigma$. Then $\pi _1(\sigma ) = {\tau }|_{c_1}(\sigma )$, and this is the configuration from Fig. 8 lower left, i.e. ${\mathbf {\varvec{\kappa }}}[\ell _2]=2$, ${\mathbf {\varvec{\kappa }}}[\ell _5]=1$ and ${\mathbf {\varvec{\kappa }}}[\ell _1]={\mathbf {\varvec{\kappa }}}[\ell _3] ={\mathbf {\varvec{\kappa }}}[\ell _4]= {\mathbf {\varvec{\kappa }}}[\ell _6] =0$. Configuration $\pi _2(\sigma ) = {\tau }|_{c_1}\cdot {\tau }|_{c_2}(\sigma )= {\tau }|_{c_2}(\pi _1(\sigma ))$ is represented on Fig. 8 lower right, i.e. ${\mathbf {\varvec{\kappa }}}[\ell _5]=3$ and all other counters are zero.

Section 5 deals with the construction of representatives of schedules that contain rules from only one looplet. Recall that construction of $\mathsf {crep}^{\varOmega }_{c_1}[\pi _0(\sigma ), {\tau }|_{c_1}]$ corresponds to the one from Example 5.2. Thus, we know that

$$\begin{aligned} \mathsf {crep}^{\varOmega }_{c_1}[\pi _0(\sigma ), {\tau }|_{c_1}]= (r_2,0), (r_3,1), (r_4,2), (r_1,2), (r_2,0), (r_3,0). \end{aligned}$$

As $c_2$ is a singleton looplet, we use the result of Sect. 5.1. Thus,

$$\begin{aligned} \mathsf {crep}^{\varOmega }_{c_2}[\pi _1(\sigma ), {\tau }|_{c_2}]= (r_5,2). \end{aligned}$$

Using the result from Sect. 5.2 we obtain that

$$\begin{aligned} \mathsf {crep}^{\varOmega }_{c_3}[\pi _2(\sigma ), {\tau }|_{c_3}]= (r_8,0),(r_7,2), \end{aligned}$$

and finaly we have the representative for $\tau$ that is

$$\begin{aligned} \mathsf {srep}_{\varOmega }[\sigma ,\tau ] =(r_2,0), (r_3,1), (r_4,2), (r_1,2), (r_2,0), (r_3,0),(r_5,2),(r_8,0),(r_7,2). \end{aligned}$$

### Lemma 6.1

(Looplet sorting) Given a threshold automaton, a context $\varOmega$, a configuration $\sigma$, a steady schedule $\tau$ applicable to $\sigma$, and a sequence $c_1, \ldots , c_m$ of all looplets in the slice ${{\mathcal R}}|_{\varOmega }$ with the property  for $1 \le i < j \le m$, the following holds:

1. Schedule ${\tau }|_{c_1}$ is applicable to the configuration $\sigma$.
2. Schedule ${\tau }|_{c_2, \ldots , c_m}$ is applicable to the configuration ${\tau }|_{c_1}(\sigma )$.
3. Schedule ${\tau }|_{c_1}\cdot \,{\tau }|_{c_2, \ldots , c_m}$, when applied to $\sigma$, results in configuration $\tau (\sigma )$.

### Proof

In the following, we show Points 1–3 one-by-one.

We need extra notation. For a local state $\ell$ we denote by $\mathbf {1}_\ell$ the $|{\mathcal L}|$-dimensional vector, where the $\ell$th component is 1, and all the other components are 0. Given a schedule $\rho = t_1 \cdots t_k$, we introduce a vector $\varDelta _{\mathbf {\varvec{\kappa }}}(\rho ) \in {\mathbb Z}^{|{\mathcal L}|}$ to keep counter difference and a vector $\varDelta _{\mathbf {g}}(\rho ) \in {\mathbb N}_0^{|\varGamma |}$ to keep difference on shared variables as follows:

$$\begin{aligned} \varDelta _{\mathbf {\varvec{\kappa }}}(\rho ) = \sum _{1 \le i \le |\rho |} t_i.{ factor }\cdot (\mathbf {1}_{t_i.{ to }} - \mathbf {1}_{t_i.{ from }}) \quad \text{ and } \quad \varDelta _{\mathbf {g}}(\rho ) = \sum _{{1\le i \le |\rho |}} t_i.\mathbf {u}\end{aligned}$$

*Proof of (1)* Assume by contradiction that schedule ${\tau }|_{c_1}$ is not applicable to configuration $\sigma$. Thus, there is a schedule $\tau '$ and a transition $t^*$ that constitute a prefix of ${\tau }|_{c_1}$, with the following property: $\tau '$ is applicable to $\sigma$, whereas $\tau ' \cdot t^*$ is not applicable to $\sigma$. Let $\ell = t^*.{ from }$ and $\ell ' = t^*.{ to }$.

There are three cases of why $t^*$ may be not applicable to $\tau '(\sigma )$:

(i) There is not enough processes to move: $(\sigma .{\mathbf {\varvec{\kappa }}}+ \varDelta _{\mathbf {\varvec{\kappa }}}(\tau ' \cdot t^*))[\ell ] < 0$. As $\tau$ is applicable to $\sigma$, there is a transition *t* of $\tau$ with $[{t.{ rule }}] \ne c_1$ and $t.{ to }= \ell$ as well as $t.{ factor }> 0$. From this, by definition of , it follows that . This contradicts the lemma’s assumption on the order .

(ii) The condition $t^*.\varphi ^{\mathrm {rise}}$ is not satisfied, that is, $\tau '(\sigma ) \not \models t^*.\varphi ^{\mathrm {rise}}$. Then, there is a guard $\varphi \in \mathsf {guard}(t^*.\varphi ^{\mathrm {rise}})$ with $\tau '(\sigma ) \not \models \varphi$.

Since $\tau$ is applicable to $\sigma$, there is a prefix $\rho \cdot t$ of $\tau$, for a schedule $\rho$ and a transition *t* that unlocks $\varphi$ in $\rho (\sigma )$, that is, $\rho (\sigma ) \not \models \varphi$ and $t(\rho (\sigma )) \models \varphi$. Thus, transition *t* changes the context: $\omega (\rho (\sigma )) \ne \omega (t(\rho (\sigma )))$. This contradicts the assumption that schedule $\tau$ is steady.

(iii) The condition $t^*.\varphi ^{\mathrm {fall}}$ is not satisfied: $\tau '(\sigma ) \not \models t^*.\varphi ^{\mathrm {fall}}$. Then, there is a guard $\varphi \in \mathsf {guard}(t^*.\varphi ^{\mathrm {fall}})$ with $\tau '(\sigma ) \not \models \varphi$.

Let $\rho$ be the longest prefix of $\tau$ satisfying ${\rho }|_{c_1} = \tau '$. Note that $\rho \cdot t^*$ is also a prefix of $\tau$. As ${\rho }|_{c_1} = \tau '$ and no transition decrements the shared variables, we conclude that $(\tau '(\sigma )).\mathbf {g}\le (\rho (\sigma )).\mathbf {g}$. From this and from the fact that $\tau '(\sigma ) \not \models \varphi$, it follows that $\rho (\sigma ) \not \models \varphi$. Thus transition $t^*$ is not applicable to $\rho (\sigma )$. This contradicts the assumption that $\tau$ is applicable to $\sigma$.

From (i), (ii), and (iii), we conclude that *(1)* holds.

*Proof of (2)* We show that ${\tau }|_{c_2, \dots , c_m}$ is applicable to ${\tau }|_{c_1}(\sigma )$.

To this end, we fix an arbitrary prefix $\tau '$ of $\tau$, a transition *t*, and a suffix $\tau ''$, that constitute $\tau$, that is, $\tau = \tau ' \cdot t \cdot \tau ''$. We show that if schedule ${\tau '}|_{c_2, \dots , c_m}$ is applicable to ${\tau }|_{c_1}(\sigma )$, then so is ${(\tau ' \cdot t)}|_{c_2, \dots , c_m}$.

Let us assume that ${\tau '}|_{c_2, \dots , c_m}$ is applicable to ${\tau }|_{c_1}(\sigma )$, and let $\sigma ''$ denote the resulting state $({\tau }|_{c_1} \cdot {\tau '}|_{c_2, \dots , c_m})(\sigma )$. We consider two cases:

- $[{t.{ rule }}] = c_1$. This case holds trivially, as ${(\tau ' \cdot t)}|_{c_2, \dots , c_m}$ equals to ${\tau '}|_{c_2, \dots , c_m}$, which is applicable to ${\tau }|_{c_1}(\sigma )$ by assumption.
- $[{t.{ rule }}] \ne c_1$. In order to prove that${(\tau ' \cdot t)}|_{c_2, \dots , c_m}$ is applicable to ${\tau }|_{c_1}(\sigma )$, we show that counters $\sigma ''.{\mathbf {\varvec{\kappa }}}$ and shared variables $\sigma ''.\mathbf {g}$ are large enough, so that transition *t* is applicable to $\sigma ''$:

(i) We start by showing that $\sigma ''.{\mathbf {\varvec{\kappa }}}[t.{ from }] \ge t.{ factor }$. We distinguish between different cases on source and target states of transition *t*.

(i.A): We will show by contradiction that there is no rule $r \in c_1$ with $t.{ to }= r.{ from }$. Let’s assume it exists. Then, on one hand, as $[{t.{ rule }}] \ne c_1$, by definition of , it follows that . On the other hand, as $[{t.{ rule }}] \ne c_1$ and $c_1, \dots , c_m$ are all classes of the rules used in $\tau$, it holds that $[{t.{ rule }}] \in \{ c_2, \dots , c_m \}$. By the lemma’s assumption, , and thus, . We arrive at a contradiction.
(i.B): Let’s consider the case of a rule $r \in c_1$ with $r.{ to }= t.{ from }$. Assume by contradiction that *t* is not applicable to $\sigma ''$, that is, $\sigma ''.{\mathbf {\varvec{\kappa }}}[t.{ from }] < t.{ factor }$. On one hand, transition *t* is not applicable to $\sigma '' = ({\tau }|_{c_1} \cdot {\tau '}|_{c_2, \dots , c_m})(\sigma )$. Then by the definition of $\varDelta _{\mathbf {\varvec{\kappa }}}$, it holds that $\sigma [t.{ from }] + (\varDelta _{\mathbf {\varvec{\kappa }}}({\tau }|_{c_1} \cdot {\tau '}|_{c_2, \dots , c_m}) + \varDelta _{\mathbf {\varvec{\kappa }}}(t))[t.{ from }] < 0$. By observing that ${\tau }|_{ c_1 } = {\tau '}|_{ c_1 } + {\tau ''}|_{ c_1 }$, we derive the following inequality:
  6.1 $$\begin{aligned}&\sigma [t.{ from }] \nonumber \\&\quad + (\varDelta _{\mathbf {\varvec{\kappa }}}({\tau '}|_{c_1}) + \varDelta _{\mathbf {\varvec{\kappa }}}({\tau ''}|_{c_1}) + \varDelta _{\mathbf {\varvec{\kappa }}}({\tau '}|_{c_2, \dots , c_m}) + \varDelta _{\mathbf {\varvec{\kappa }}}(t))[t.{ from }] < 0 \end{aligned}$$
  On the other hand, schedule $\tau = \tau ' \cdot t \cdot \tau ''$ is applicable to configuration $\sigma$. Thus, $\sigma [t.{ from }] + (\varDelta _{\mathbf {\varvec{\kappa }}}(\tau ') + \varDelta _{\mathbf {\varvec{\kappa }}}(t) + \varDelta _{\mathbf {\varvec{\kappa }}}(\tau ''))[t.{ from }] \ge 0$. By observing that ${\tau }|_{ c_1 } = {\tau '}|_{ c_1 } + {\tau ''}|_{ c_1 }$ and ${\tau }|_{c_2, \dots , c_m} = {\tau '}|_{c_2, \dots , c_m} + {\tau ''}|_{c_2, \dots , c_m}$, we arrive at:
  6.2 $$\begin{aligned}&\sigma [t.{ from }] + (\varDelta _{\mathbf {\varvec{\kappa }}}({\tau '}|_{c_1}) + \varDelta _{\mathbf {\varvec{\kappa }}}({\tau '}|_{c_2, \dots , c_m}) \nonumber \\&\quad + \varDelta _{\mathbf {\varvec{\kappa }}}(t) + \varDelta _{\mathbf {\varvec{\kappa }}}({\tau ''}|_{c_1}) + \varDelta _{\mathbf {\varvec{\kappa }}}({\tau ''}|_{c_2, \dots , c_m}))[t.{ from }] \ge 0 \end{aligned}$$
  By subtracting (6.2) from (6.1), and by commutativity of vector addition, we arrive at $\varDelta _{\mathbf {\varvec{\kappa }}}({\tau ''}|_{c_2, \dots , c_m})[t.{ from }] > 0$. Thus, there is a transition $t'$ in ${\tau ''}|_{c_2, \dots , c_m}$ and a rule $r' \in c_1$ such that $t'.{ to }= r'.{ from }$. We again arrived at the contradictory Case (i.A). Hence, transition *t* must be applicable to configuration $\sigma ''$.
(i.C): Otherwise, neither $t.{ from }$ nor $t.{ to }$ belong to the set of local states affected by the rules from $c_1$, i.e., $\{t.{ from }, t.{ to }\} \cap \{ \ell \mid \exists r \in c_1.\ r.{ from }= \ell \vee r.{ to }= \ell \}$ is empty. Then, schedule ${\tau }|_{c_1}$ does not change the counter ${\mathbf {\varvec{\kappa }}}[t.{ from }]$, and $\varDelta _{\mathbf {\varvec{\kappa }}}(\tau ')[t.{ from }] = \varDelta _{\mathbf {\varvec{\kappa }}}({\tau '}|_{c_2, \dots , c_m})[t.{ from }]$. As *t* is applicable to $\tau '(\sigma )$, that is, $(\tau '(\sigma )).{\mathbf {\varvec{\kappa }}}[t.{ from }] \ge t.{ factor }$, we conclude that $\sigma ''.{\mathbf {\varvec{\kappa }}}[t.{ from }] \ge t.{ factor }$.

(ii) We now show that $\sigma '' \models t.\varphi ^{\mathrm {rise}}\wedge t.\varphi ^{\mathrm {fall}}$. Assume by contradiction that $\sigma '' \not \models t.\varphi ^{\mathrm {rise}}\wedge t.\varphi ^{\mathrm {fall}}$. There are two cases to consider.

If $$\sigma '' \not \models t.\varphi ^{\mathrm {rise}}$$.
By definition, the shared variables are never decremented in a non-singleton looplet. As $\tau '$ is a prefix of $\tau$, schedule ${\tau }|_{c_1} \cdot {\tau '}|_{c_2, \dots , c_m}$ includes all transitions of $\tau '$. Thus, $\varDelta _{\mathbf {g}}({\tau }|_{c_1} \cdot {\tau '}|_{c_2, \dots , c_m}) \ge \varDelta _{\mathbf {g}}(\tau ')$. From this and $\sigma '' \not \models t.\varphi ^{\mathrm {rise}}$, it follows that $\tau '(\sigma ) \not \models t.\varphi ^{\mathrm {rise}}$. This contradicts applicability of $\tau$ to $\sigma$.
If $$\sigma '' \not \models t.\varphi ^{\mathrm {fall}}$$.
Then, there is a guard $\varphi \in \mathsf {guard}(t.\varphi ^{\mathrm {fall}})$ with $\tau ''(\sigma ) \not \models \varphi$. On one hand, ${\tau }|_{c_1} \cdot {\tau '}|_{c_2, \dots , c_m}$ is applicable to $\sigma$. On the other hand, $\tau$ is applicable to $\sigma$. We notice that $\varDelta _{\mathbf {g}}(\tau ) = \varDelta _{\mathbf {g}}({\tau }|_{c_1}) + \varDelta _{\mathbf {g}}({\tau '}|_{c_2,\dots ,c_m}) + \varDelta _{\mathbf {g}}({\tau ''}|_{c_2,\dots ,c_m}) + \varDelta _{\mathbf {g}}(t) \ge \varDelta _{\mathbf {g}}({\tau }|_{c_1}) + \varDelta _{\mathbf {g}}({\tau '}|_{c_2,\dots ,c_m})$. As shared variables are never decreased, it follows that $({\tau }|_{c_1} \cdot {\tau '}|_{c_2,\dots ,c_m})(\sigma ) \not \models \varphi$. Thus, $\omega (\sigma ) \ne \omega (\tau (\sigma ))$. This contradicts the assumption on that schedule $\tau$ is steady.

Having proved that, we conclude that transition *t* is applicable to configuration $({\tau }|_{c_1} \cdot {\tau '}|_{c_2, \dots , c_m})(\sigma )$. Thus, by induction $({\tau }|_{c_1} \cdot {\tau }|_{c_2, \dots , c_m})(\sigma )$ is applicable to $\sigma$. We conclude that Point *2* of the theorem holds.

*Proof of (3)* By the commutativity property of vector addition,

$$\begin{aligned} \varDelta _{\mathbf {\varvec{\kappa }}}({\tau }|_{c_1} \cdot {\tau }|_{c_2, \dots , c_m}) = \varDelta _{\mathbf {\varvec{\kappa }}}({\tau }|_{c_1}) + \varDelta _{\mathbf {\varvec{\kappa }}}({\tau }|_{c_2, \dots , c_m}) = \sum _{1 \le i \le |\tau |} \varDelta _{\mathbf {\varvec{\kappa }}}(t_i) = \varDelta _{\mathbf {\varvec{\kappa }}}(\tau ). \end{aligned}$$

Thus, $({\tau }|_{c_1} \cdot {\tau }|_{c_2, \dots , c_m})(\sigma ) = \tau (\sigma )$, and Point (3) follows.

We have thus shown all three points of Lemma 6.1. $\square$

### Proof

(*of Theorem* 6.1) By iteratively applying Lemma 6.1, we prove by induction that schedule ${\tau }|_{c_1} \cdot \ldots \cdot {\tau }|_{c_m}$ is applicable to $\sigma$ and results in $\tau (\sigma )$. From Theorem 5.1, we conclude that each schedule ${\tau }|_{c_i}$ can be replaced by its representative $\mathsf {crep}^{\varOmega }_{c_i}[\pi _{i-1}(\sigma ), {\tau }|_{c_i}]$. Thus, $\mathsf {srep}_{\varOmega }[\sigma ,\tau ]$ is applicable to $\sigma$ and results in $\tau (\sigma )$. By Proposition 3.4, schedule $\mathsf {srep}_{\varOmega }[\sigma ,\tau ]$ is steady, since $\omega (\sigma ) = \omega (\tau (\sigma ))$. $\square$

Finally, we show that for a given context, there is a schema that generates all paths of such representative schedules.

### Theorem 6.2

Fix a threshold automaton and a context $\varOmega$. Let $c_1, \ldots , c_m$ be the sorted sequence of all looplets of the slice ${{\mathcal R}}|_{\varOmega }$, i.e., . Schema $\mathsf {sschema}_{\varOmega }{} = \mathsf {cschema}^{\varOmega }_{c_1} \circ \cdots \circ \mathsf {cschema}^{\varOmega }_{c_m}$ has two properties: (a) For a configuration $\sigma$ with $\omega (\sigma ) = \varOmega$ and a steady schedule $\tau$ applicable to $\sigma$, $\textsf {path}(\sigma , \tau ')$ of the representative $\tau '=\mathsf {srep}_{\varOmega }[\sigma ,\tau ]$ is generated by $\mathsf {sschema}_{\varOmega }$; and (b) the length of $\mathsf {sschema}_{\varOmega }$ is at most $2\cdot |({{\mathcal R}}|_{\varOmega })|$.

### Proof

Fix a configuration $\sigma$ with $\omega (\sigma ) = \varOmega$ and a steady schedule $\tau$ applicable to $\sigma$. As $\mathsf {srep}_{\varOmega }[\sigma ,\tau ]$ is a sorted sequence of the looplet representatives, all paths of $\mathsf {srep}_{\varOmega }[\sigma ,\tau ]$ are generated by $\mathsf {sschema}_{\varOmega }$, which is not longer than $2\cdot |({{\mathcal R}}|_{\varOmega })|$. $\square$

## Proving the main result

Using the results from Sects. 5 and 6, for each configuration and each schedule (without restrictions) we construct a representative schedule.

### Theorem 7.1

Given a threshold automaton, a configuration $\sigma$, and a schedule $\tau$ applicable to $\sigma$, there exists a schedule $\mathsf {rep}[\sigma ,\tau ]$ with the following properties:

- $\mathsf {rep}[\sigma ,\tau ]$ is applicable to $\sigma$, and $\mathsf {rep}[\sigma ,\tau ](\sigma ) = \tau (\sigma )$,
- $$|\mathsf {rep}[\sigma ,\tau ]| \le 2 \cdot |{\mathcal R}| \cdot (|\varPhi ^{\mathrm {rise}}| + |\varPhi ^{\mathrm {fall}}| + 1) + |\varPhi ^{\mathrm {rise}}| + |\varPhi ^{\mathrm {fall}}|$$.

### Proof

Given a threshold automaton, fix a configuration $\sigma$ and a schedule $\tau$ applicable to $\sigma$. Let $\varOmega _1, \ldots , \varOmega _{K+1}$ be the maximal monotonically increasing sequence of contexts such that $\textsf {path}(\sigma , \tau )$ is consistent with the sequence by Definition 3.7. From Proposition 3.2, the length of the sequence is $K+1 = |\varPhi ^{\mathrm {rise}}| + |\varPhi ^{\mathrm {fall}}|+1$. Thus, there are at most *K* transitions $t^\star _{1}, \ldots , t^\star _{K}$ in $\tau$ that change their context, i.e., for $i \in \{1,\dots ,K\}$, it holds $\omega (\sigma _i) \sqsubset \omega (t^\star _{i}(\sigma _i))$ for $t^\star _{i}$’s respective state $\sigma _i$ in $\tau$. Therefore, we can divide $\tau$ into $K+1$ steady schedules separated by the transitions $t^\star _{1},\ldots ,t^\star _{K}$:

$$\begin{aligned} \tau = \nu _1 \cdot t^\star _{1} \cdot \nu _2 \cdots \nu _K \cdot t^\star _{K} \cdot \nu _{K+1}. \end{aligned}$$

Now, the main idea is to replace the steady schedules with their representatives from Theorem 6.1. That is, using $t^\star _{1}, \ldots , t^\star _{K}$ and $\nu _{1}, \ldots , \nu _{K+1}$, we construct the schedules $\rho _1, \ldots , \rho _K$ (by convention, $\rho _0$ is the empty schedule):

$$\begin{aligned} \rho _i = \rho _{i-1} \cdot \nu _i \cdot t^\star _{i} \quad \text{ for } 1 \le i \le K. \end{aligned}$$

Finally, the representative schedule $\mathsf {rep}[\tau ,\sigma ]$ is constructed as follows:

$$\begin{aligned} \mathsf {rep}_{\varOmega _1}[\sigma ,\nu _1] \cdot t^\star _{1} \cdot \mathsf {rep}_{\varOmega _2}[\rho _1(\sigma ),\nu _2] \cdots \mathsf {rep}_{\varOmega _K}[\rho _{K-1}(\sigma ),\nu _K] \cdot t^\star _{K} \cdot \mathsf {rep}_{\varOmega _{K+1}}[\rho _K(\sigma ),\nu _{K+1}] \end{aligned}$$

From Theorem 6.1, it follows that $\mathsf {rep}[\tau ,\sigma ]$ is applicable to $\sigma$ and it results in $\tau (\sigma )$. Moreover, the representative of a steady schedule is not longer than $2 |{\mathcal R}|$, which together with *K* transitions gives us the bound $2|{\mathcal R}| (K+1) + K$. As we have that $K =|\varPhi ^{\mathrm {rise}}| + |\varPhi ^{\mathrm {fall}}|$, this gives us the required bound. $\square$

Further, given a maximal monotonically increasing sequence *z* of contexts, we construct a schema that generates all paths of the schedules consistent with *z*:

### Theorem 7.2

For a threshold automaton and a monotonically increasing sequence *z* of contexts, there exists a schema $\mathsf {schema}(z)$ that generates all paths of the representative schedules that are consistent with *z*, and the length of $\mathsf {schema}(z)$ does not exceed $3 \cdot |{\mathcal R}| \cdot (|\varPhi ^{\mathrm {rise}}| + |\varPhi ^{\mathrm {fall}}|) + 2 \cdot |{\mathcal R}|$.

### Proof

Given a threshold automaton, let $\rho _{\mathsf {all}}$ be the sequence $r_1, \ldots , r_{|{\mathcal R}|}$ of all rules from ${\mathcal R}$, and let $z=\varOmega _0, \ldots , \varOmega _m$ be a monotonically increasing sequence of contexts. By the construction in Theorem 7.1, each representative schedule $\mathsf {rep}[\sigma ,\tau ]$ consists of the representatives of steady schedules terminated with transitions that change the context. Then, for each context $\varOmega _i$, for $0 \le i < m$, we compose $\mathsf {sschema}_{\varOmega }$ and $\{\varOmega _i\}\,\rho _{\mathsf {all}}\,\{\varOmega _{i+1}\}$. This composition generates the representative of a steady schedule and the transition changing the context from $\varOmega _i$ to $\varOmega _{i+1}$. Consequently, we construct the $\mathsf {schema}(z)$ as follows:

$$\begin{aligned} (\mathsf {sschema}_{\varOmega _0} \,\circ \,\{\varOmega _0\}\,\rho _{\mathsf {all}}\,\{\varOmega _1\}) \circ \ldots \circ (\mathsf {sschema}_{\varOmega _{m-1}} \,\circ \,\{\varOmega _{m-1}\}\,\rho _{\mathsf {all}}\,\{\varOmega _{m}\}) \,\circ \,\mathsf {sschema}_{\varOmega _{m}} \end{aligned}$$

By inductively applying Theorem 6.2, we prove that $\mathsf {schema}(z)$ generates all paths of schedules $\mathsf {rep}[\sigma ,\tau ]$ that are consistent with the sequence *z*. We get the needed bound on the length of $\mathsf {schema}(z)$ by using an argument similar to Theorem 7.1 and by noting that for every context, instead of one rule that is changing it, we add $|{\mathcal R}|$ extra rules. $\square$

## Complete set of schemas and optimizations

Our proofs show that the set of schemas is easily computed from the TA: the threshold guards are syntactic parts of the TA, and enable us to directly construct increasing sequences of contexts. To find a slice of the TA for a given context, we filter the rules with unlocked guards, i.e., check whether the context contains the guard. To produce the simple schema of a looplet, we compute a spanning tree over the slice. To construct simple schemas, we do a topological sort over the looplets. For example, it takes just 30 s to compute the schemas in our longest experiment that runs for 4 h. In our tool we have implemented the following optimizations that lead to simpler and fewer SMT queries.

*Entailment optimization* We say that a guard $\varphi _1 \in \varPhi ^{\mathrm {rise}}$ *entails* a guard $\varphi _2 \in \varPhi ^{\mathrm {rise}}$, if for all combinations of parameters $\mathbf {p}\in \mathbf {P}_{RC}$ and shared variables $\mathbf {g}\in {\mathbb N}_0^{|\varGamma |}$, it holds that $(\mathbf {g}, \mathbf {p}) \models \varphi _1 \rightarrow \varphi _2$. For instance, in our example, $\varphi _3 :y \ge (2t + 1) - f$ entails $\varphi _2 :y \ge (t + 1) - f$. If $\varphi _1$ entails $\varphi _2$, then we can omit all monotonically increasing sequences that contain a context $(\varOmega ^\mathrm {rise}, \varOmega ^{\mathrm {fall}})$ with $\varphi _1 \in \varOmega ^\mathrm {rise}$ and $\varphi _2 \not \in \varOmega ^\mathrm {rise}$. If the number of schemas before applying this optimization is *m*! and there are *k* entailments, then the number of schemas reduces from *m*! to $(m-k)!$. A similar optimization is introduced for the guards from $\varPhi ^{\mathrm {fall}}$.

*Control flow optimization* Based on the proof of Lemma 6.1, we introduce the following optimization for TAs that are directed acyclic graphs (possibly with self loops). We say that a rule $r \in {\mathcal R}$ *may unlock* a guard $\varphi \in \varPhi ^{\mathrm {rise}}$, if there is a $\mathbf {p}\in \mathbf {P}_{RC}$ and $\mathbf {g}\in {\mathbb N}_0^{{|\varGamma |}}$ satisfying: $(\mathbf {g}, \mathbf {p}) \models r.\varphi ^{\mathrm {rise}}\wedge r.\varphi ^{\mathrm {fall}}$ (the rule is unlocked); $(\mathbf {g}, \mathbf {p}) \not \models \varphi$ (the guard is locked); $(\mathbf {g}+ r.\mathbf {u}, \mathbf {p}) \models \varphi$ (the guard is now unlocked).

In our example from Fig. 2, the rule $r_1 : true \mapsto x{\texttt {++}}$ may unlock the guard $\varphi _1:x \ge \lceil (n+t)/2 \rceil - f$.

Let $\varphi \in \varPhi ^{\mathrm {rise}}$ be a guard, $r'_1, \dots , r'_m$ be the rules that use $\varphi$, and $r_1, \dots , r_k$ be the rules that may unlock $\varphi$. If , for $1 \le i \le k$ and $1 \le j \le m$, then we exclude some sequences of contexts as follows (we call $\varphi$ *forward-unlockable*). Let $\psi _1, \dots , \psi _n \in \varPhi ^{\mathrm {rise}}$ be the guards of $r_1, \dots , r_k$. Guard $\varphi$ cannot be unlocked before $\psi _1, \dots , \psi _n$, and thus we can omit all sequences of contexts, where $\varphi$ appears in the contexts before $\psi _1, \dots , \psi _n$. Moreover, as $\psi _1, \dots , \psi _n$ are the only guards of the rules unlocking $\varphi$, we omit the sequences with different combinations of contexts involving $\varphi$ and the guards from $\varPhi ^{\mathrm {rise}}{\setminus } \{\varphi , \psi _1, \dots , \psi _n\}$. Finally, as the rules $r'_1, \dots , r'_m$ appear after the rules $r_1, \dots , r_k$ in the order , the rules $r'_1, \dots , r'_m$ appear after the rules $r_1, \dots , r_k$ in a rule sequence of every schema. Thus, we omit the combinations of the contexts involving $\varphi$ and $\psi _1, \dots , \psi _n$.

Hence, we add all forward-unlockable guards to the initial context (we still check the guards of the rules in the SMT encoding in Sect. 9). If the number of schemas before applying this optimization is *m*! and there are *k* forward-unlocking guards, then the number of schemas reduces from *m*! to $(m-k)!$. A similar optimization is introduced for the guards from $\varPhi ^{\mathrm {fall}}$.

## Checking a schema with SMT

We decompose a schema into a sequence of simple schemas, and encode the simple schemas. Given a simple schema $S=\{\varOmega _1\}\,r_1, \dots , r_m\,\{\varOmega _2\}$, which contains *m* rules, we construct an SMT formula such that every model of the formula represents a path from $\mathcal{L}(S)$ —the language of paths generated by schema *S* —and for every path in $\mathcal{L}(S)$ there is a corresponding model of the formula. Thus, we need to model a path of $m+1$ configurations and *m* transitions (whose acceleration factors may be 0).

To represent a configuration $\sigma _i$, for $0 \le i \le m$, we introduce two vectors of SMT variables: Given the set of local states ${\mathcal L}$ and the set of shared variables $\varGamma$, a vector $\mathbf {k}^i = (k^i_1, \dots , k^i_{|{\mathcal L}|})$ to represent the process counters, a vector $\mathbf {x}^i = (x^i_1, \dots , x^i_{|\varGamma |})$ to represent the shared variables. We call the pair $(\mathbf {k}^i, \mathbf {x}^i)$ the *layer* *i*, for $1 \le i \le m$.

Based on this we encode schemas, for which the sequence of rules $r_1, \dots , r_m$ is fixed. We exploit this in two ways: First, we encode for each layer *i* the constraints of rule $r_i$. Second, as this constraint may update only two counters —the processes move from and move to according to the rule —we do not need $|{\mathcal L}|$ counter variables per layer, but only encode the two counters per layer that have actually changed. As is a common technique in bounded model checking, the counters that are not changed are “reused” from previous layers in our encoding. By doing so, we encode the schema rules with $|{\mathcal L}| + |\varGamma | + m \cdot (2 + |\varGamma |)$ integer variables, 2*m* equations, and inequalities in linear integer arithmetic that represent threshold guards that evaluate to true (at most the number of threshold guards times *m* of these inequalities).

In the following, we use the notation $[k:m]$ to denote the set $\{k, \dots , m\}$. In order to reuse the variables from the previous layers, we introduce a function $\upsilon : {\mathcal L}\times [0:m] \rightarrow [0:m]$ that for a layer $i \in [0:m]$ and a local state $\ell \in {\mathcal L}$, gives the largest number $j \le i$ of the layer, where the counter $k^j_\ell$ is updated:

$$\begin{aligned} \upsilon (\ell , i) = {\left\{ \begin{array}{ll} i, &{} \quad \text{ if } \, i=0 \vee \ell \in \{ r_i.{ from }, r_i.{ to }\}\\ \upsilon (\ell , i - 1), &{}\quad \text{ otherwise }. \end{array}\right. } \end{aligned}$$

Having defined layers, we encode: the effect of rules on counters and shared variables (in formulas *M* and *U* below), the effect of rules on the configuration (*T*), restrictions imposed by contexts (*C*), and, finally, the reachability question.

To represent *m* transitions, for each transition $i \in [1:m]$, we introduce a non-negative variable $\delta ^i$ for the acceleration factor, and define two formulas: formula $M^\ell (i-1,i)$ to express the update of the counter of local state $\ell \in {\mathcal L}$, and formula $U^x(i-1,i)$ to represent the update of the shared variable $x \in \varGamma$:

$$\begin{aligned} M^\ell (i-1,i) \;&\equiv \; {\left\{ \begin{array}{ll} k^i_\ell = k^{\upsilon (\ell , i-1)}_\ell + \delta ^i, &{}\quad \text{ for } \ell = r_i.{ to } \text{ and } i \in [1:m] \\ k^i_\ell = k^{\upsilon (\ell , i-1)}_\ell - \delta ^i, &{}\quad \text{ for } \ell = r_i.{ from } \text{ and } i \in [1:m] \\ true , &{} \quad \text{ otherwise } \end{array}\right. } \\ U^x(i-1,i \; )&\equiv \; {\left\{ \begin{array}{ll} x^i = x^{i-1} + \delta ^i \cdot u, &{}\quad \text{ if } \,u = r_i.\mathbf {u}[j] > 0,\\ true , &{}\quad \text{ otherwise }. \end{array}\right. } \end{aligned}$$

The formula $T(i-1,i)$ collects all constraints by the rule $r_i$:

$$\begin{aligned} T(i-1,i) \; \equiv \; \bigwedge _{\ell \in {\mathcal L}} M^\ell (i-1,i) \wedge \bigwedge _{x \in \varGamma } U^x(i-1,i). \end{aligned}$$

For a formula $\varphi$, we denote by $\varphi [\mathbf {x}^i]$ the formula, where each variable $x \in \varGamma$ is substituted with $x^i$. Then, given a context $\varOmega =(\varOmega ^\mathrm {rise}, \varOmega ^{\mathrm {fall}})$, a formula $C^{\varOmega }(i)$ adds the constraints of the context $\varOmega$ on the layer *i*:

$$\begin{aligned} C_{\varOmega }(i) \; \equiv \; \bigwedge _{\varphi \in \varOmega ^\mathrm {rise}} \varphi [\mathbf {x}^i] \wedge \bigwedge _{\varphi \in \varPhi ^{\mathrm {rise}}{\setminus } \varOmega ^\mathrm {rise}} \lnot \varphi [\mathbf {x}^i] \wedge \bigwedge _{\varphi \in \varOmega ^{\mathrm {fall}}} \lnot \varphi [\mathbf {x}^i] \wedge \bigwedge _{\varphi \in \varPhi ^{\mathrm {fall}}{\setminus } \varOmega ^{\mathrm {fall}}} \varphi [\mathbf {x}^i]. \end{aligned}$$

Finally, the formula $C_{\varOmega _1}(0) \wedge T(0, 1) \wedge \dots \wedge T(m-1,m) \wedge C_{\varOmega _2}(m)$ captures all the constraints of the schema $S=\{\varOmega _1\}\,r_1, \dots , r_m\,\{\varOmega _2\}$, and thus, its models correspond to the paths of schedules that are generated by *S*.

Let *I*(0) be the formula over the variables of layer *i* that captures the initial states of the threshold automaton, and *B*(*i*) be a state property over the variables of layer *i*. Then, parameterized reachability for the schema *S* is encoded with the following formula in linear integer arithmetic:

$$\begin{aligned} I(0) \wedge C_{\varOmega _1}(0) \wedge T(0, 1) \wedge \dots \wedge T(m-1,m) \wedge C_{\varOmega _2}(m) \wedge \big (B(0) \vee \dots \vee B(m)\big ). \end{aligned}$$

## Experiments

We have extended our tool ByMC (Byzantine Model Checker [2]) with the technique discussed in this paper. All of our benchmark algorithms were originally published in pseudo-code, and we model them in a parametric extension of Promela, which was discussed in [27, 34].

### Benchmarks

We revisited several asynchronous FTDAs that were evaluated in [33, 41]. In addition to these classic FTDAs, we considered asynchronous (Byzantine) consensus algorithms, namely, BOSCO [57], C1CS [10], and CF1S [18], that are designed to work despite partial failure of the distributed system. In contrast to the conference version of this paper [39], we used a new version of the benchmarks from [37] that have been slightly updated for liveness properties. Hence, for some benchmarks, the running times of our tool may vary from [39]. The benchmarks, their source code in parametric of Promela, and the code of the threshold automata are freely available [30].

### Implementation

ByMC supports several tool chains (shown in Fig. 1, p. 3), the first using counter abstraction (that is, process counters over an abstract domain), and the second using counter systems with counters over integers:

*Data and counter abstractions* In this chain, the message counters are first mapped to parametric intervals, e.g., counters range over the abstract domain $\hat{D}=\{[0, 1)$, $[1, t+1), [t+1, n-t), [n-t, \infty )\}$. By doing so, we obtain a finite (data) abstraction of each process, and thus we can represent the system as a counter system: We maintain one counter $\kappa [\ell ]$ per local state $\ell$ of a process, as well as the counters for the sent messages. Then, in the counter abstraction step, every process counter $\kappa [\ell ]$ is mapped to the set of parametric intervals $\hat{D}$. As the abstractions may produce spurious counterexamples, we run them in an abstraction-refinement loop that incrementally prunes spurious transitions and unfair executions. More details on the data and counter abstractions and refinement can be found in [33]. In our experiments, we use two kinds of model checkers as backend:

1. *BDD* The counter abstraction is checked with nuXmv [11] using Binary Decision Diagrams (BDDs). For safety properties, the tool executes the command check_invar. In our experiments, we used the timeout of 3 days, as there was at least one benchmark that needed a bit more than a day to complete.
2. *BMC* The counter abstraction is checked with nuXmv using bounded model checking [6]. To ensure completeness (at the level of counter abstraction), we explore the computations of the length up to the diameter bounds that were obtained in [41]. To efficiently eliminate shallow spurious counterexamples, we first run the bounded model checker in the incremental mode up to length of 30. This is done by issuing the nuXmv command check_ltlspec_sbmc_inc, which uses the built-in SAT solver MiniSAT. Then, we run a single-shot SAT problem by issuing the nuXmv command gen_ltlspec_sbmc and checking the generated formula with the SAT solver lingeling [5]. In our experiments, we set the timeout to 1 day.

*Reachability for threshold automata* In this tool chain, to obtain a threshold automaton, our tool first applies data abstraction over the domain $\hat{D}$ to the Promela code, which abstracts the message counters that keep the number of messages received by every process, while the message counters for the sent messages are kept as integers. More details can be found in [40]. Having constructed a threshold automaton, we compare two verification approaches:

1. ${{ PARA}}^{2}$ *Bounded model checking with SMT* The approach of this article. BYMC enumerates the schemas (as explained in Sect. 4), encodes them in SMT (as explained in Sect. 9) and checks every schema with the SMT solver Z3 [17].
2. *FAST* *Acceleration of counter automata* In this chain, our tool constructs a threshold automaton and checks the reachability properties with the existing tool FAST [3]. For comparison with our tool, we run FAST with the MONA plugin that produced the best results in our experiments.

The challenge in the verification of FTDAs is the immense non-determinism caused by interleavings, asynchronous message passing, and faults. In our modeling, all these are reflected in non-deterministic choices in the Promela code. To obtain threshold automata, as required for our technique, our tool constructs a parametric interval data abstraction [33] that adds to non-determinism.

Comparing to [39], in this paper, we have introduced an optimization to schema checking that dramatically reduced the running times for some of the benchmarks. In this optimization, we group schemas in a prefix tree, whose nodes are contexts and edges are simple schemas. In each node of the prefix tree, our tool checks, whether there are configurations that are reachable from the initial configurations by following the schemas in the prefix. If there are no such reachable configurations, we can safely prune the whole suffix and thus prove many schemas to be unsatisfiable at once.

### Evaluation

Table 1 summarizes the features of threshold automata that are automatically constructed by ByMC from parametric Promela. The number of local states $|{\mathcal L}|$ varies from 7 (FRB and STRB) to hundreds (C1CS and CBC). Our threshold automata are obtained by applying interval abstraction to Promela code, which keeps track of the number of messages received by each process. Thus, the number $|{\mathcal L}|$ is proportional to the number of control states and $|\widehat{D}|^k$, where $\widehat{D}$ is the domain of parametric intervals (discussed above) and *k* is the number of message types. Sometimes, one can manually construct a more efficient threshold automaton that models the same fault-tolerant distributed algorithm and preserves the same safety properties. For instance, Fig. 2 shows a manual abstraction of ABA that has only 5 local states, in contrast to 61 local states in the automatic abstraction (cf. Table 1). We leave open the question of whether one can automatically construct a minimal threshold automaton with respect to given specifications.

**Table 1:** The benchmarks used in our experiments. Some benchmarks, e.g., ABA, require us to consider several cases on the parameters, which are mentioned in the column “Case”. The meaning of the other columns is as follows: $|{\mathcal L}|$ is the number of local states in TA, $|{\mathcal R}|$ is the number of rules in TA, $|\varPhi ^{\mathrm {rise}}|$ and $|\varPhi ^{\mathrm {fall}}|$ is the number of (R)- and (F)-guards respectively. Finally, $|\mathcal{S}|$ is the number of enumerated schemas, and Bound is the theoretical upper bound on $|\mathcal{S}|$, as given in Theorem 4.2

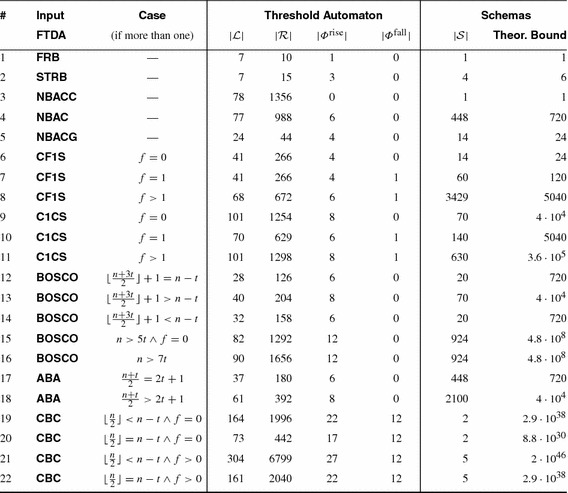

**Table 2:** Summary of our experiments on AMD Opteron®6272, 32 cores, 192 GB. The symbols are: “” for timeout (72 h. for BDD and 24 h. otherwise); “” for memory overrun of 32 GB; “” for BDD nodes overrun; “” for timeout in the refinement loop (72 h. for BDD and 24 h. otherwise); “” for spurious counterexamples due to counter abstraction

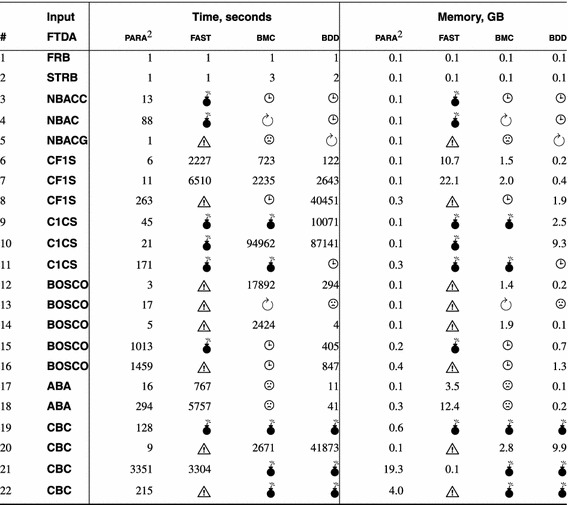	

Table 2 summarizes our experiments conducted with the techniques introduced in Sect. 10.2: BDD, BMC, PARA$^2$, and FAST. On large problems, our new technique works significantly better than BDD- and SAT-based model checking. BDD-based model checking works very well on top of counter abstraction. Importantly, our new technique does not use abstraction refinement. In comparison to our earlier experiments [39], we verified safety of a larger set of benchmarks with nuXmv. We believe that this is due to the improvements in nuXmv and, probably, slight modifications of the benchmarks from [37].

NBAC and NBACC are challenging as the model checker produces many spurious counterexamples, which are an artifact of counter abstraction losing or adding processes. When using SAT-based model checking, the individual calls to nuXmv are fast, but the abstraction-refinement loop times out, due to a large number of refinements (about 500). BDD-based model checking times out when looking for a counterexample. Our new technique, preserves the number of proceses, and thus, there are no spurious counterexamples of this kind. In comparison to the general-purpose acceleration tool FAST, our tool uses less memory and is faster on the benchmarks where FAST is successful.

As predicted by the distributed algorithms literature, our tool finds counterexamples, when we relax the resilience condition. In contrast to counter abstraction, our new technique gives us concrete values of the parameters and shows how many processes move at each step of the counterexample.

Our new method uses integer counters and thus does not introduce spurious behavior due to counter abstraction, but still has spurious behavior due to data abstraction on complex FTDAs such as BOSCO, C1CS, and NBAC. In these cases, we manually refine the interval domain by adding new symbolic interval borders, see [33]. We believe that these intervals can be obtained directly from threshold automata, and no refinement is necessary. We leave this question to future work.

*Sets of schemas and time to check a single schema*

On one hand, Theorem 4.2 gives us a theoretical bound on the number of schemas to be explored. On the other hand, optimizations discussed in Sect. 8 introduce many ways of reducing the number of schemas. Two columns in Table 1 compare the theoretical bound and the practical number of schemas: the column “Theoretical bound” shows the bound of $(|\varPhi ^{\mathrm {rise}}|+|\varPhi ^{\mathrm {fall}}|)!$, while the column $|\mathcal{S}|$ shows the actual number of schemas. (For reachability, we are merging the schemas with the prefix tree, and thus the actual number of explored schemas is even smaller.) As one can see, the theoretical bound is quite pessimistic, and is only useful to show completeness of the set of schemas. The much smaller numbers for the fault-tolerant distributed algorithms are due to a natural order on guards, e.g., as $x \ge t+1$ becomes true earlier than $x \ge n - t$ under the resilience condition $n>3t$. The drastic reduction in the case of CBC is due to the control flow optimization discussed in Sect. 8 and the fact that basically all guards are forward-unlocking.

**Fig. 9:**
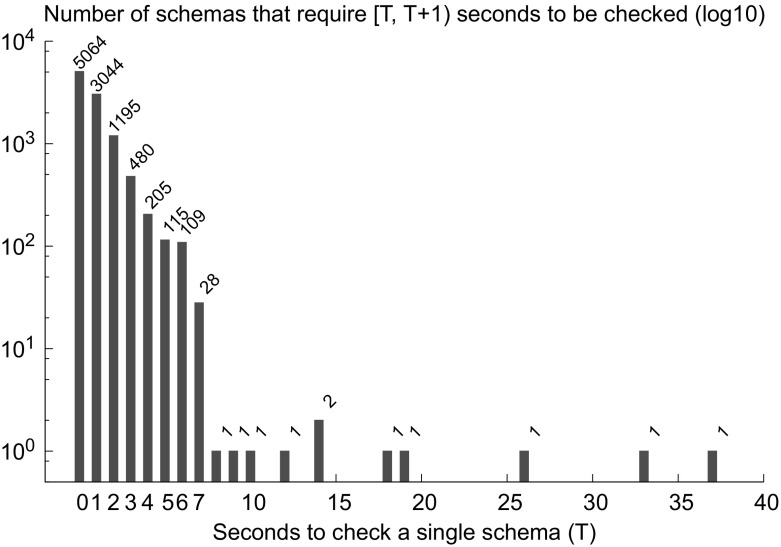
The times required to check individual schemas and the distribution of schemas over these times (the value 0 refers to the running times of less than a second). The benchmarks containing the schemas that are verified in (a) $T \ge 8$ sec. and (b) $T \ge 18$ sec. are: (a) C1CS, CBC, CF1S, and (b) CBC and CF1S

When doing experiments, we noticed that the only kinds of guards that cannot be treated by our optimizations and blow up the number of schemas are the guards that use independent shared variables. For instance, consider the guards $x_0 \ge n - t$ and $x_1 \ge n - t$ that are counting the number of 0’s and 1’s sent by the correct processes. Even though they are mutually exlusive under the resilience condition $n > 3t$, our tool has to explore all possible orderings of these guards. We are not aware of a reduction that would prevent our method from exploding in the number of schemas for this example.

Since the schemas can be checked independently, one can check them in parallel. Figure 9 shows a distribution of schemas along with the time needed to check an individual schema. There are only a few divergent schemas that required more than 7 s to get checked, while the large portion of schemas require 1–3 s. Hence, a parallel implementation of the tool should verify the algorithms significantly faster. We leave such a parallel extension for future work.

## Discussions and related work

We introduced a method to efficiently check reachability properties of FTDAs in a parameterized way. If $n>7t$ as for BOSCO, even the simplest interesting case with $t=2$ leads to a system size that is out of range of explicit state model checking. Hence, FTDAs force us to develop parameterized verification methods.

The problem we consider is concerned with parameterized model checking, for which many interesting results exist [14, 15, 21–23, 35]; cf. [7] for a survey. However, the FTDAs considered by us run under the different assumptions.

From a methodological viewpoint, our approach combines techniques from several areas including compact programs [49], counter abstraction [4, 55], completeness thresholds for bounded model checking [6, 16, 42], partial order reduction [8, 28, 53, 59], and Lipton’s movers [48]. Regarding counter automata, our result entails *flattability* [46] of every counter system of threshold automata: a complete set of schemas immediately gives us a flat counter automaton. Hence, the acceleration-based semi-algorithms [3, 46] should in principle terminate on the systems of TAs, though it did not always happen in our experiments. Similar to our SMT queries based on schemas, the *inductive data flow graphs* iDFG introduced in [24] are a succinct representations of schedules (they call them traces) for systems where the number of processes (or threads) is fixed. The work presented in [25] then considers parameterized verification. Further, our execution schemas are inspired by a general notion of *semi-linear path schemas* SLPS [45, 46]. We construct a small complete set of schemas and thus a provably small SLPS. Besides, we distinguish counter systems and counter abstraction: the former counts processes as integers, while the latter uses counters over a finite abstract domain, e.g., $\{0,1, many \}$ [55].

Many distributed algorithms can be represented with I/O Automata [50] or TLA+ [44]. In these frameworks, correctness is typically shown with a proof assistant, while model checking is used as a debugger on small instances. Parameterized model checking is not a concern there, except one notable result [32].

The results presented in this article can be used to check reachability properties of FTDAs. We can thus establish safety of FTDAs. However, for fault-tolerant distributed algorithms liveness is as important as safety: The seminal impossibility result by Fischer, Lynch, and Paterson [26] states that a fault-tolerant consensus algorithm cannot ensure both safety and liveness in asynchronous systems. In recent work [37] we also considered liveness verification, or more precisely, verification of temporal logic specification with the ${\textsf {G}}\,$ and ${\textsf {F}}\,$ temporal operators. In [37], we use the results of this article as a black box and show that combinations of schemas can be used to generate counterexamples to liveness properties, and that we can verify both safety and liveness by complete SMT-based bounded model checking.

## Footnotes

1 Our model requires *all* variables to be non-negative integers. Although these constraints (e.g., $x^1 \ge 0$) have to be encoded in the SMT queries, we omit these constraints here for a more concise presentation.
